# Supplementary material for: Contribution of behavioural variability to representational drift
Source: eLife. 2022 Aug 30;11:e77907. doi: 10.7554/eLife.77907 (PMC9481246; doi:10.7554/eLife.77907)
Supplement: Supplementary file 1. [file elife-77907-supp1.docx]

### **Supplementary Table 1: Information of recording sessions in different datasets.**

| session id | session type | age (day) | sex | genotype | pupilometry |
| --- | --- | --- | --- | --- | --- |
| 715093703 | brain observatory | 118 | M | Sst-IRES-Cre x Ai32 | NO |
| 719161530 | brain observatory | 122 | M | Sst-IRES-Cre x Ai32 | NO |
| 721123822 | brain observatory | 125 | M | Pvalb-IRES-Cre x Ai32 | NO |
| 732592105 | brain observatory | 100 | M | WT | NO |
| 737581020 | brain observatory | 108 | M | WT | NO |
| 739448407 | brain observatory | 112 | M | WT | NO |
| 742951821 | brain observatory | 120 | M | WT | YES |
| 743475441 | brain observatory | 121 | M | WT | YES |
| 744228101 | brain observatory | 122 | M | WT | YES |
| 746083955 | brain observatory | 98 | F | Pvalb-IRES-Cre x Ai32 | YES |
| 750332458 | brain observatory | 91 | M | WT | YES |
| 750749662 | brain observatory | 92 | M | WT | YES |
| 751348571 | brain observatory | 93 | F | Vip-IRES-Cre x Ai32 | YES |
| 754312389 | brain observatory | 140 | M | WT | YES |
| 754829445 | brain observatory | 141 | M | WT | YES |
| 755434585 | brain observatory | 100 | M | Vip-IRES-Cre x Ai32 | YES |
| 756029989 | brain observatory | 96 | M | Sst-IRES-Cre x Ai32 | YES |
| 757216464 | brain observatory | 105 | M | WT | YES |
| 757970808 | brain observatory | 106 | M | WT | YES |
| 758798717 | brain observatory | 102 | M | Sst-IRES-Cre x Ai32 | YES |
| 759883607 | brain observatory | 113 | M | WT | YES |
| 760345702 | brain observatory | 103 | M | Pvalb-IRES-Cre x Ai32 | YES |
| 760693773 | brain observatory | 110 | F | Sst-IRES-Cre x Ai32 | YES |
| 761418226 | brain observatory | 119 | M | WT | YES |
| 762120172 | brain observatory | 100 | M | Vip-IRES-Cre x Ai32 | YES |
| 762602078 | brain observatory | 110 | M | Sst-IRES-Cre x Ai32 | YES |
| 763673393 | brain observatory | 126 | M | WT | YES |
| 773418906 | brain observatory | 124 | F | Pvalb-IRES-Cre x Ai32 | YES |
| 791319847 | brain observatory | 116 | M | Vip-IRES-Cre x Ai32 | YES |
| 797828357 | brain observatory | 107 | M | Pvalb-IRES-Cre x Ai32 | YES |
| 798911424 | brain observatory | 110 | F | Vip-IRES-Cre x Ai32 | YES |
| 799864342 | brain observatory | 129 | M | WT | YES |
| 766640955 | functional connectivity | 133 | M | WT | YES |
| 767871931 | functional connectivity | 135 | M | WT | YES |
| 768515987 | functional connectivity | 136 | M | WT | NO |
| 771160300 | functional connectivity | 142 | M | WT | YES |
| 771990200 | functional connectivity | 108 | M | WT | YES |
| 774875821 | functional connectivity | 114 | M | WT | YES |
| 778240327 | functional connectivity | 120 | M | WT | YES |
| 778998620 | functional connectivity | 121 | M | WT | YES |
| 779839471 | functional connectivity | 122 | M | WT | YES |
| 781842082 | functional connectivity | 126 | M | WT | YES |
| 786091066 | functional connectivity | 111 | F | Sst-IRES-Cre x Ai32 | YES |
| 787025148 | functional connectivity | 114 | M | Sst-IRES-Cre x Ai32 | YES |
| 789848216 | functional connectivity | 119 | M | Sst-IRES-Cre x Ai32 | YES |
| 793224716 | functional connectivity | 120 | M | WT | YES |
| 794812542 | functional connectivity | 120 | F | Sst-IRES-Cre x Ai32 | YES |
| 816200189 | functional connectivity | 128 | F | Vip-IRES-Cre x Ai32 | YES |
| 819186360 | functional connectivity | 128 | F | WT | YES |
| 819701982 | functional connectivity | 135 | F | Vip-IRES-Cre x Ai32 | YES |
| 821695405 | functional connectivity | 134 | F | WT | YES |
| 829720705 | functional connectivity | 112 | M | Pvalb-IRES-Cre x Ai32 | YES |
| 831882777 | functional connectivity | 137 | M | Sst-IRES-Cre x Ai32 | YES |
| 835479236 | functional connectivity | 121 | M | Vip-IRES-Cre x Ai32 | YES |
| 839068429 | functional connectivity | 129 | F | Sst-IRES-Cre x Ai32 | YES |
| 839557629 | functional connectivity | 115 | M | Pvalb-IRES-Cre x Ai32 | YES |
| 840012044 | functional connectivity | 116 | M | Pvalb-IRES-Cre x Ai32 | NO |
| 847657808 | functional connectivity | 126 | F | WT | YES |
