# Supplementary figures and images for: Contribution of behavioural variability to representational drift
Source: eLife. 2022 Aug 30;11:e77907. doi: 10.7554/eLife.77907 (PMC9481246; doi:10.7554/eLife.77907)

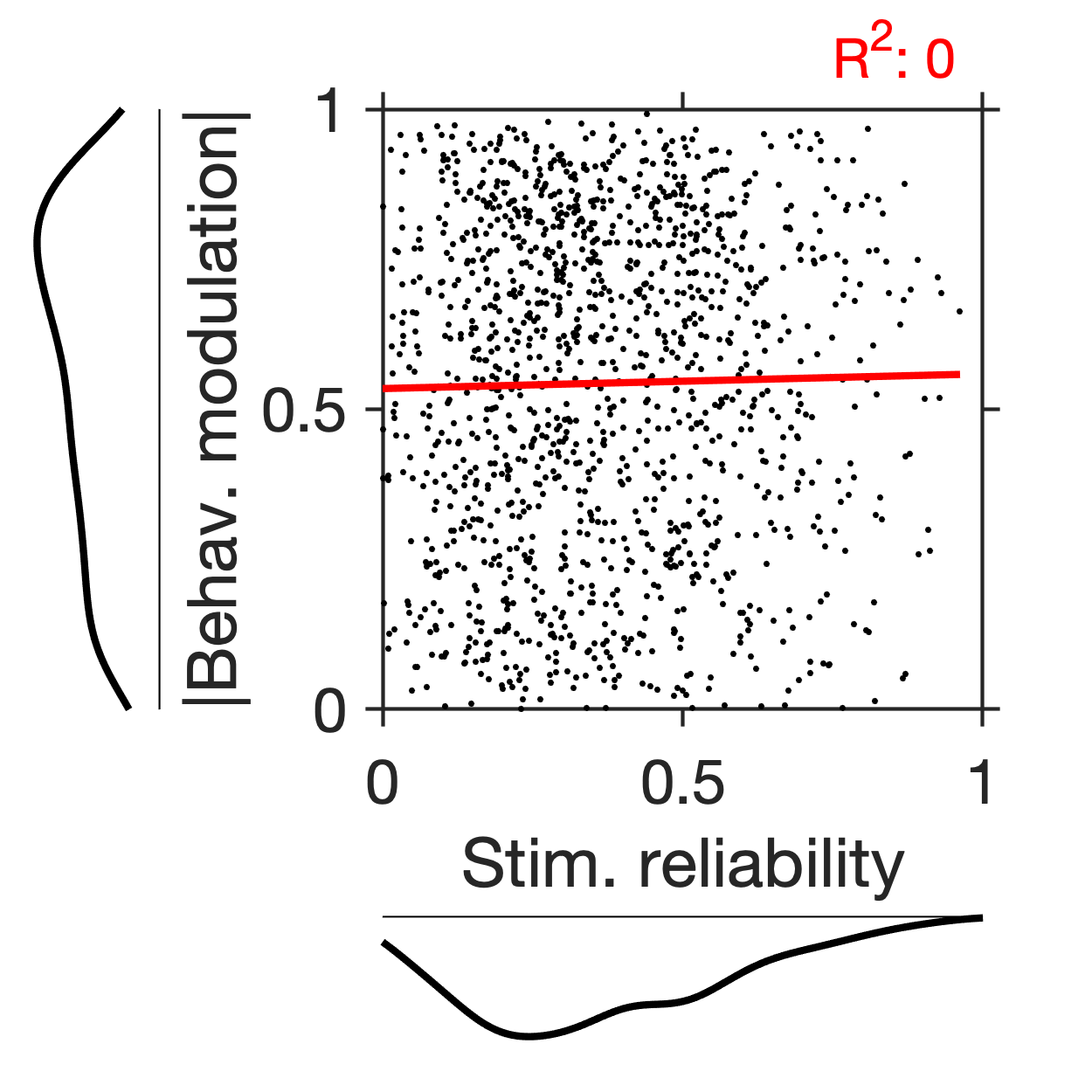

Supplement: Source code 1. [file elife-77907-code1.zip › Codes_eLife/Figure2/scatter_hist_2.png]

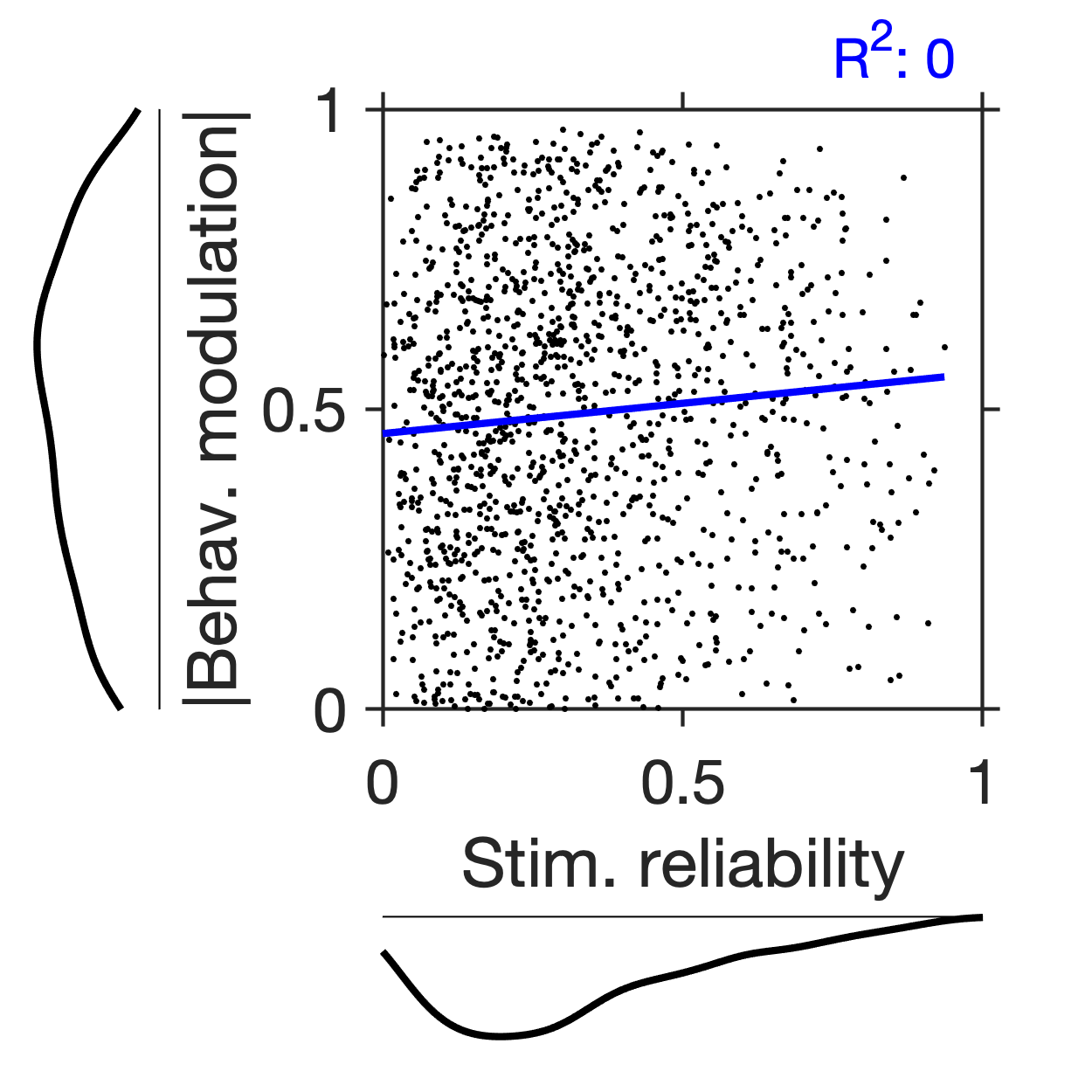

Supplement: Source code 1. [file elife-77907-code1.zip › Codes_eLife/Figure2/scatter_hist_1.png]

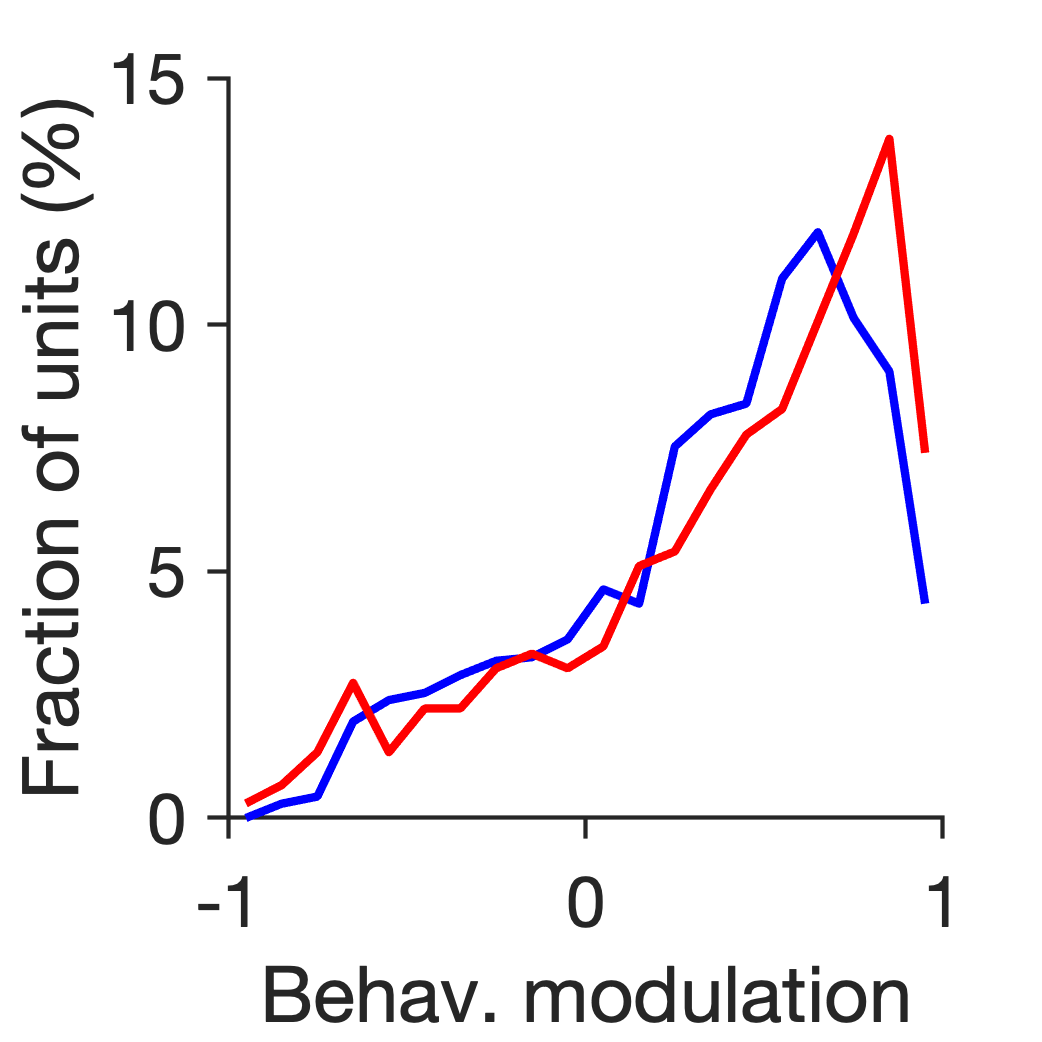

Supplement: Source code 1. [file elife-77907-code1.zip › Codes_eLife/Figure2/CC_data_ccw_dist.png]

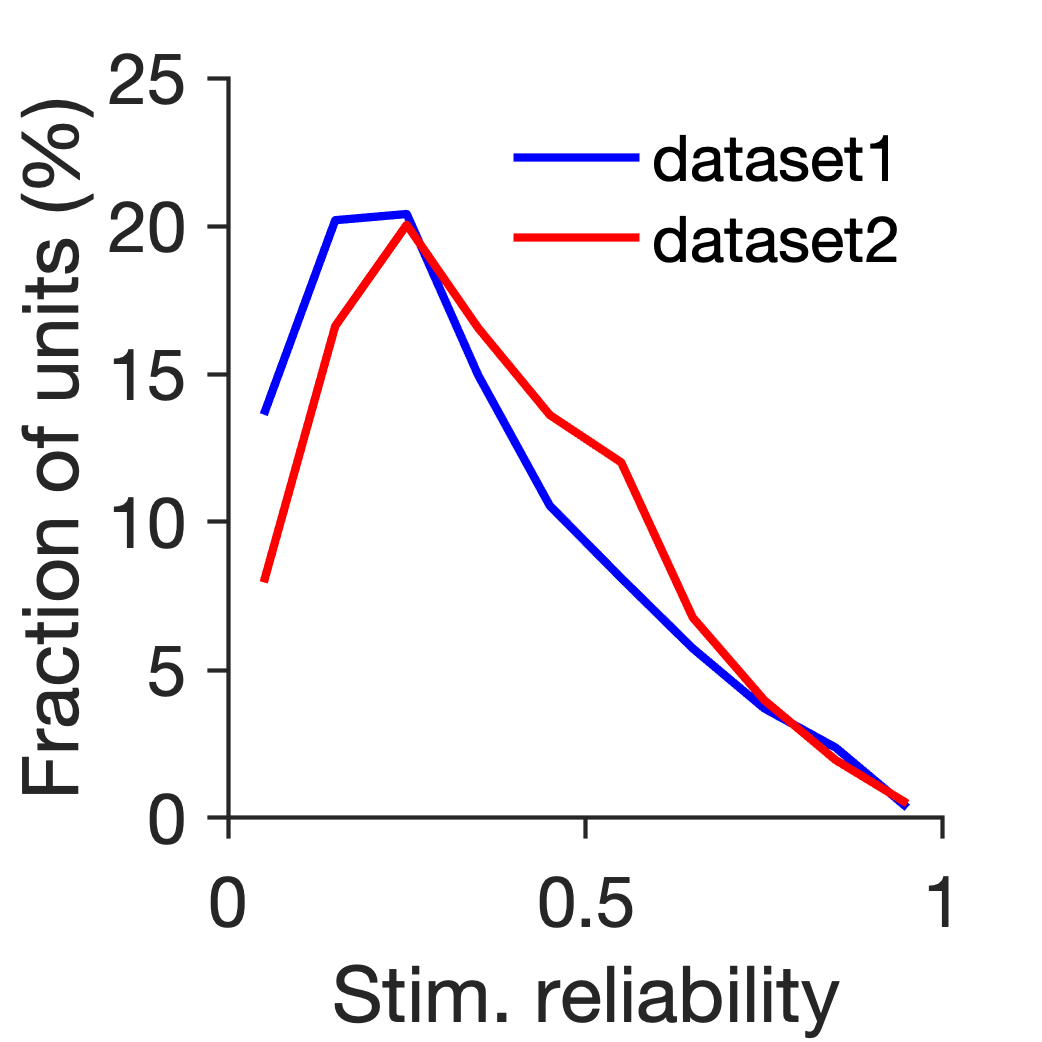

Supplement: Source code 1. [file elife-77907-code1.zip › Codes_eLife/Figure2/CC_data_ccr_dist.png]

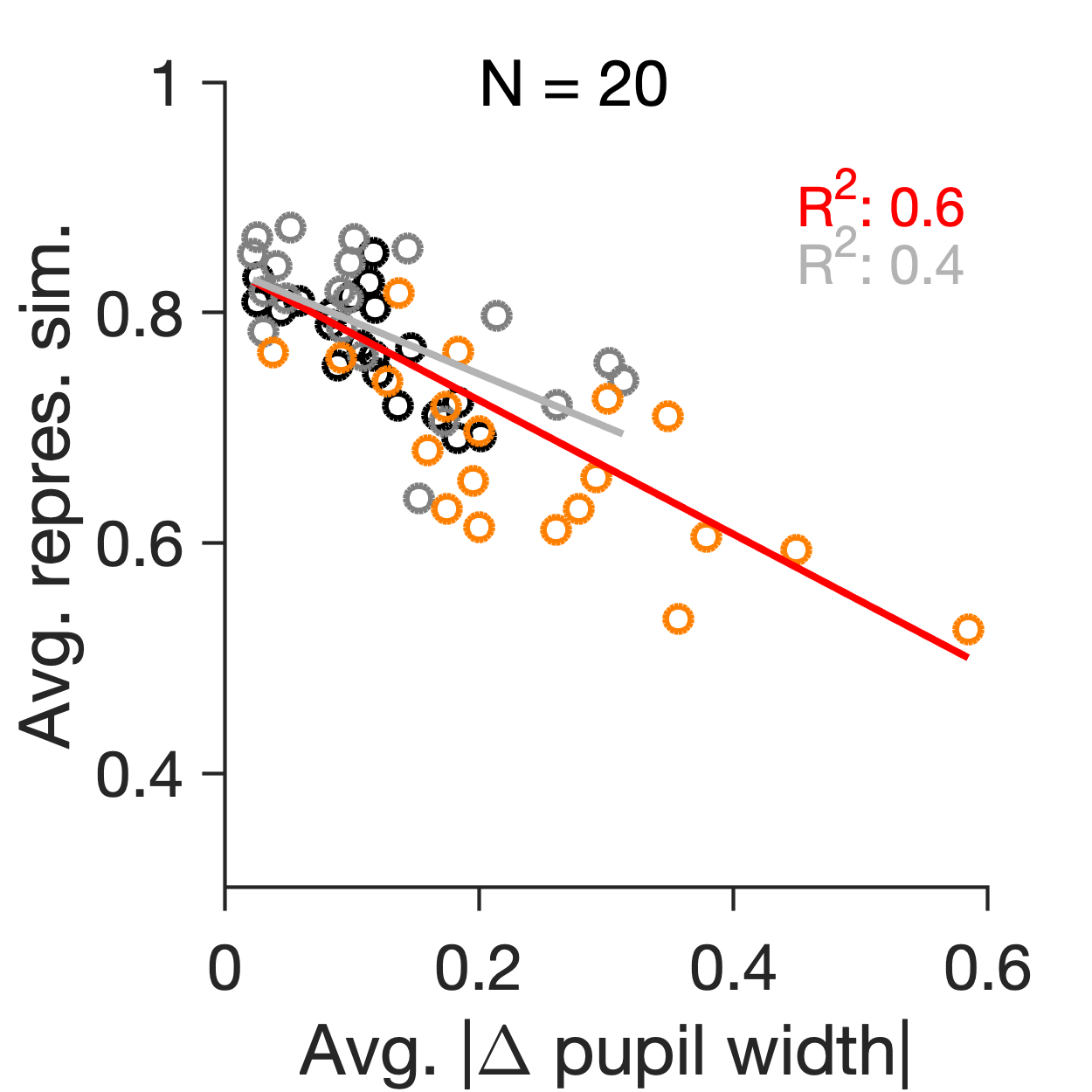

Supplement: Source code 1. [file elife-77907-code1.zip › Codes_eLife/Figure1/VISp__repDrift_ppl_avg_brain_observatory_1.1.png]

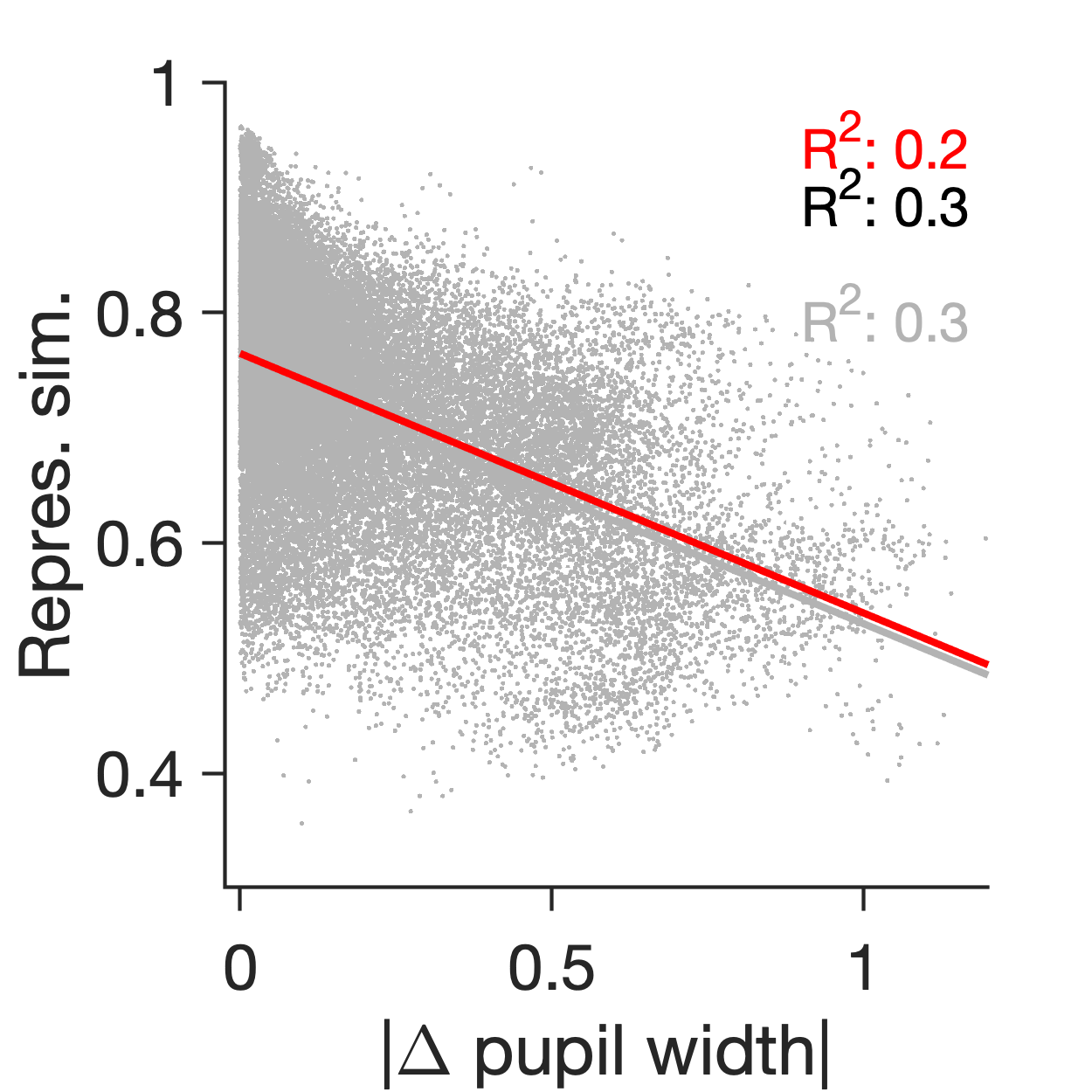

Supplement: Source code 1. [file elife-77907-code1.zip › Codes_eLife/Figure1/VISp__repDrift_ppl_all_functional_connectivity.png]

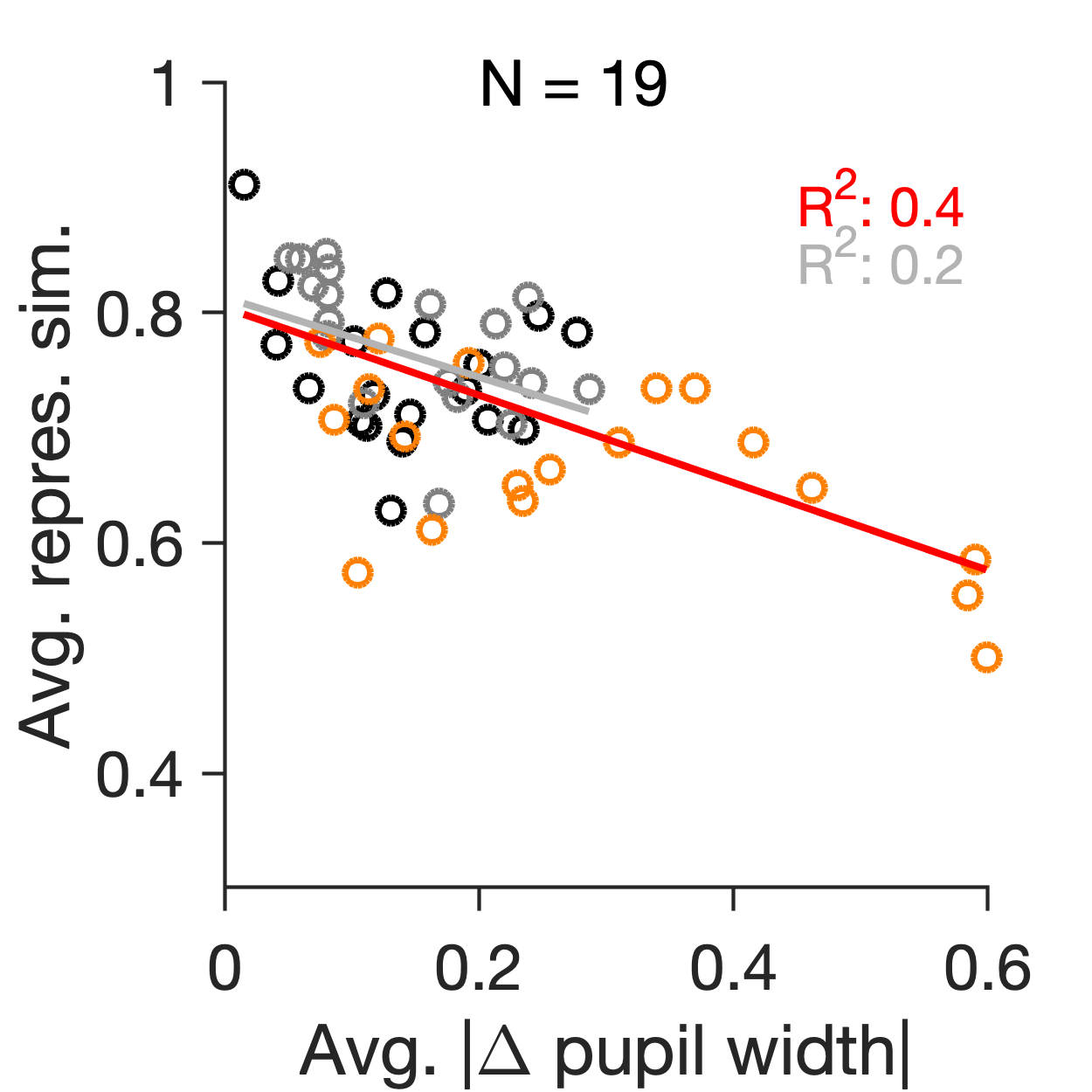

Supplement: Source code 1. [file elife-77907-code1.zip › Codes_eLife/Figure1/VISp__repDrift_ppl_avg_functional_connectivity.png]

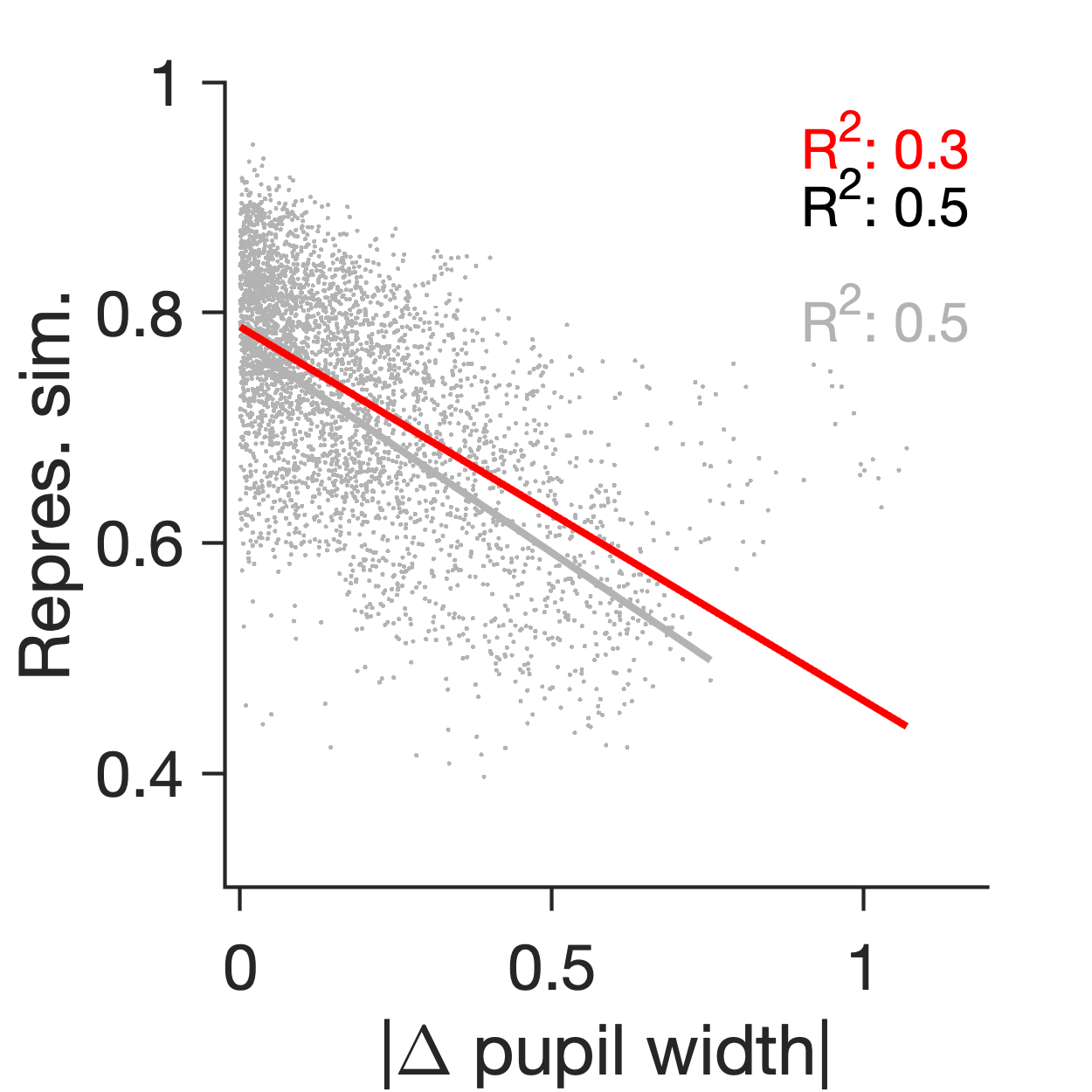

Supplement: Source code 1. [file elife-77907-code1.zip › Codes_eLife/Figure1/VISp__repDrift_ppl_all_brain_observatory_1.1.png]

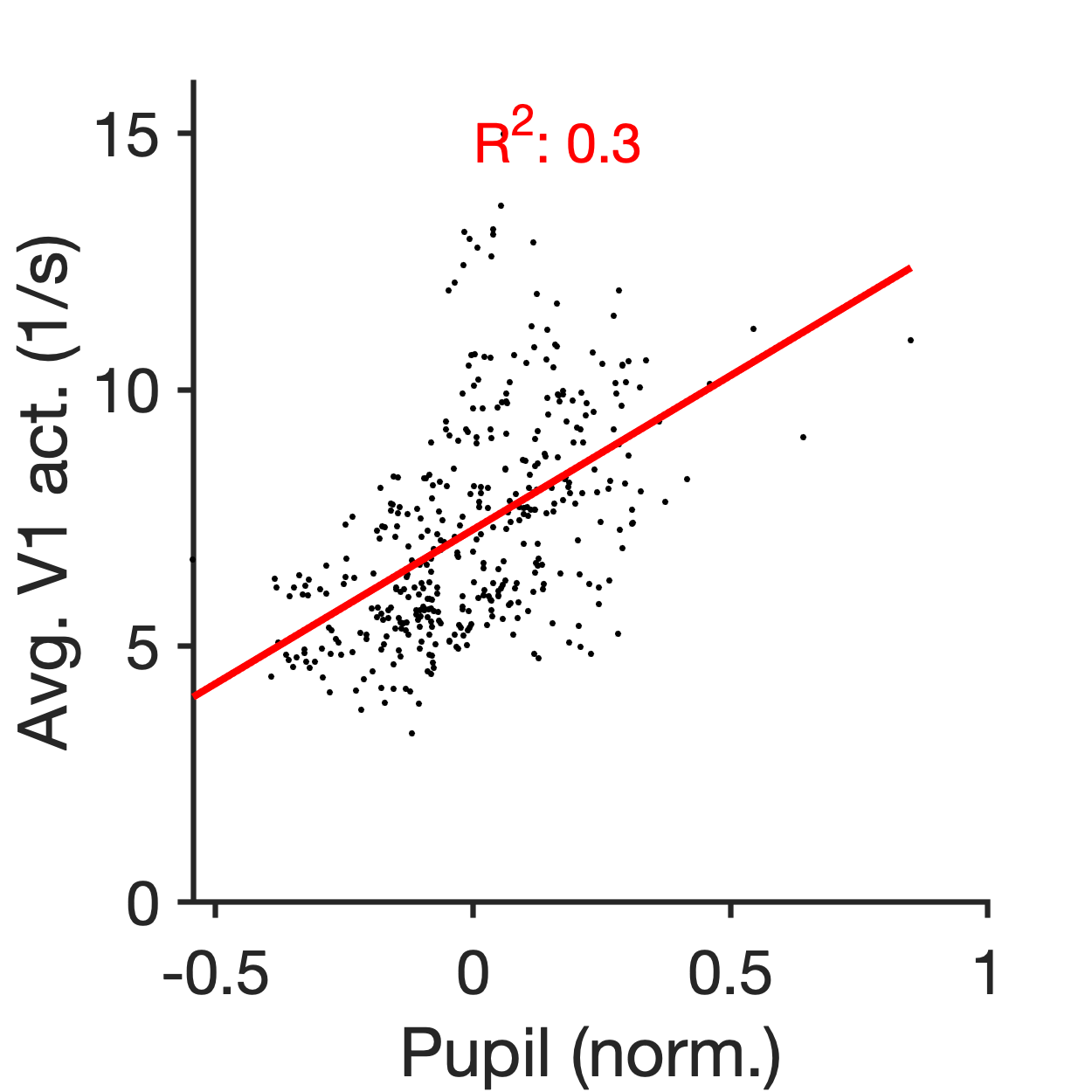

Supplement: Source code 1. [file elife-77907-code1.zip › Codes_eLife/Figure4/All/brain_observatory_1.1_VISp_pplAct_all.png]

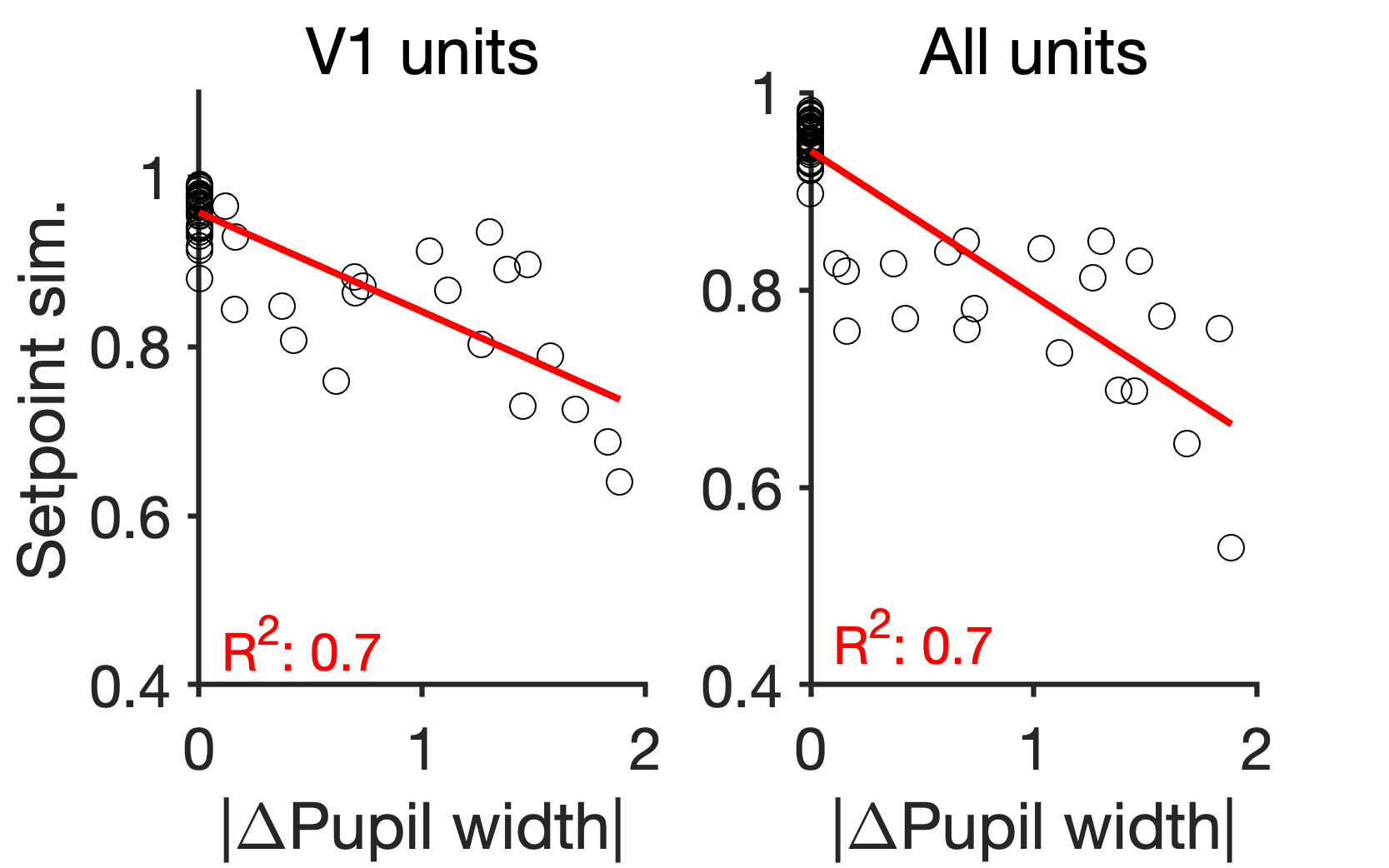

Supplement: Source code 1. [file elife-77907-code1.zip › Codes_eLife/Figure4/All/brain_observatory_1.1_VISp_SetSim_ppd_all.png]

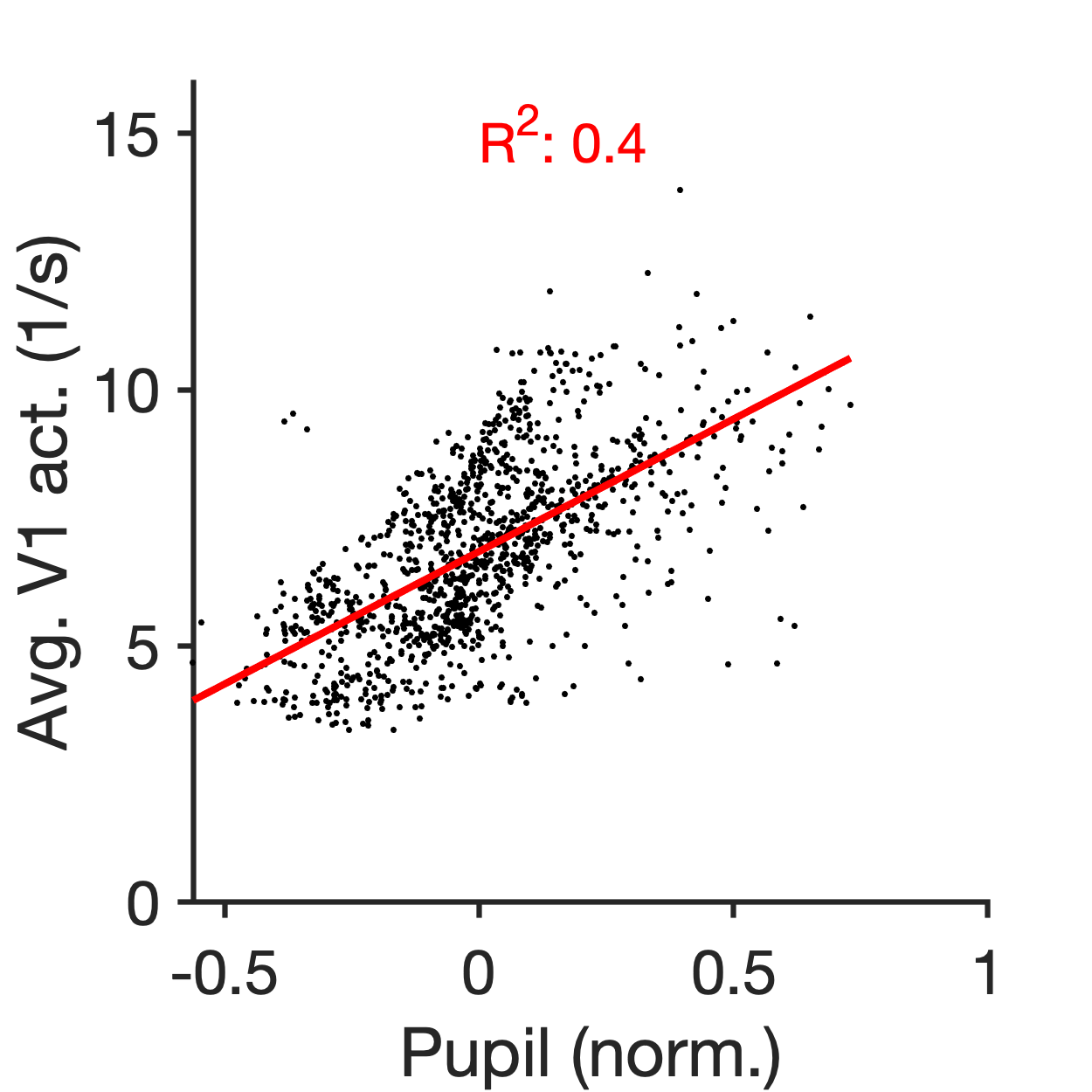

Supplement: Source code 1. [file elife-77907-code1.zip › Codes_eLife/Figure4/All/functional_connectivity_VISp_pplAct_all.png]

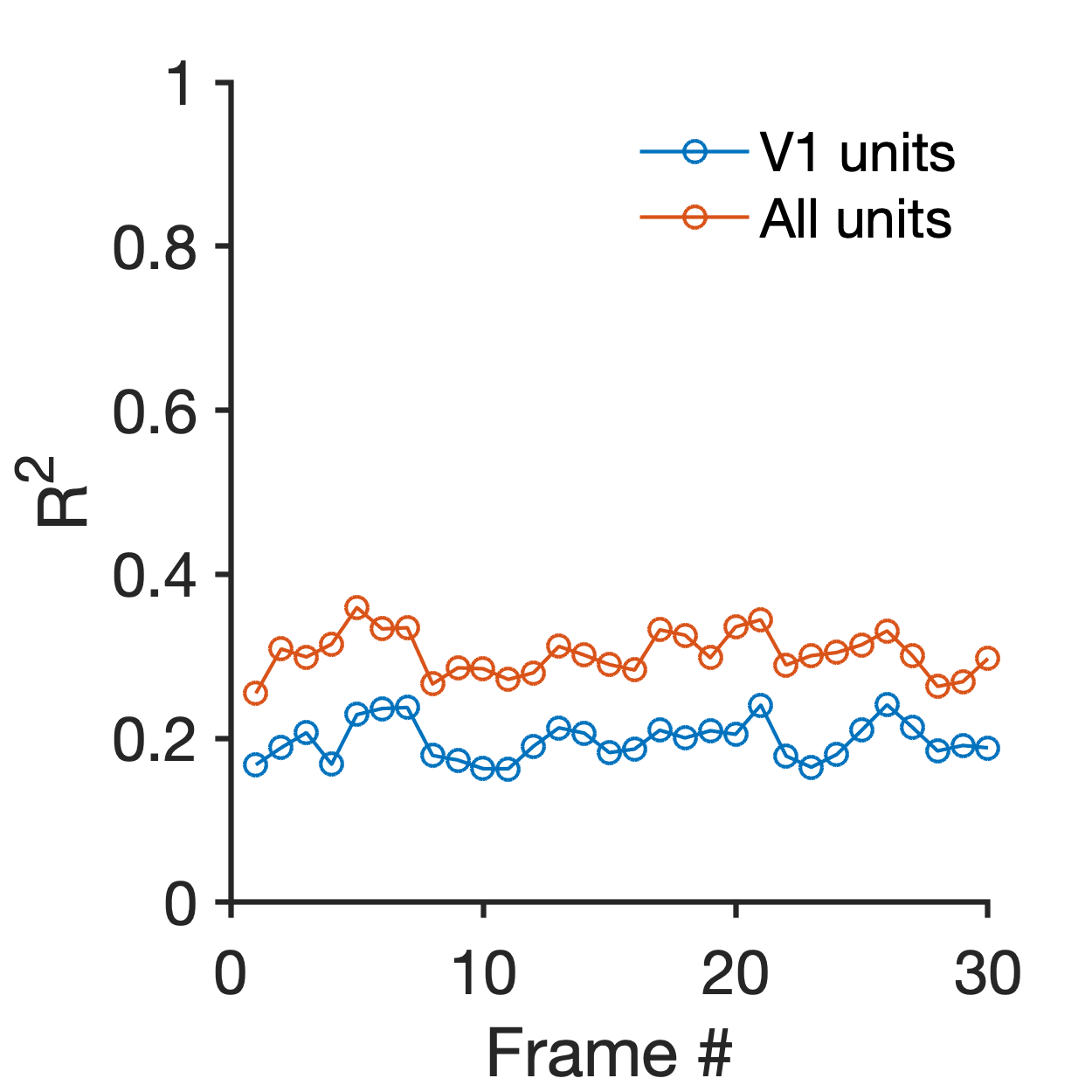

Supplement: Source code 1. [file elife-77907-code1.zip › Codes_eLife/Figure4/All/brain_observatory_1.1_VISp_SetSim_ppd_stimWise.png]

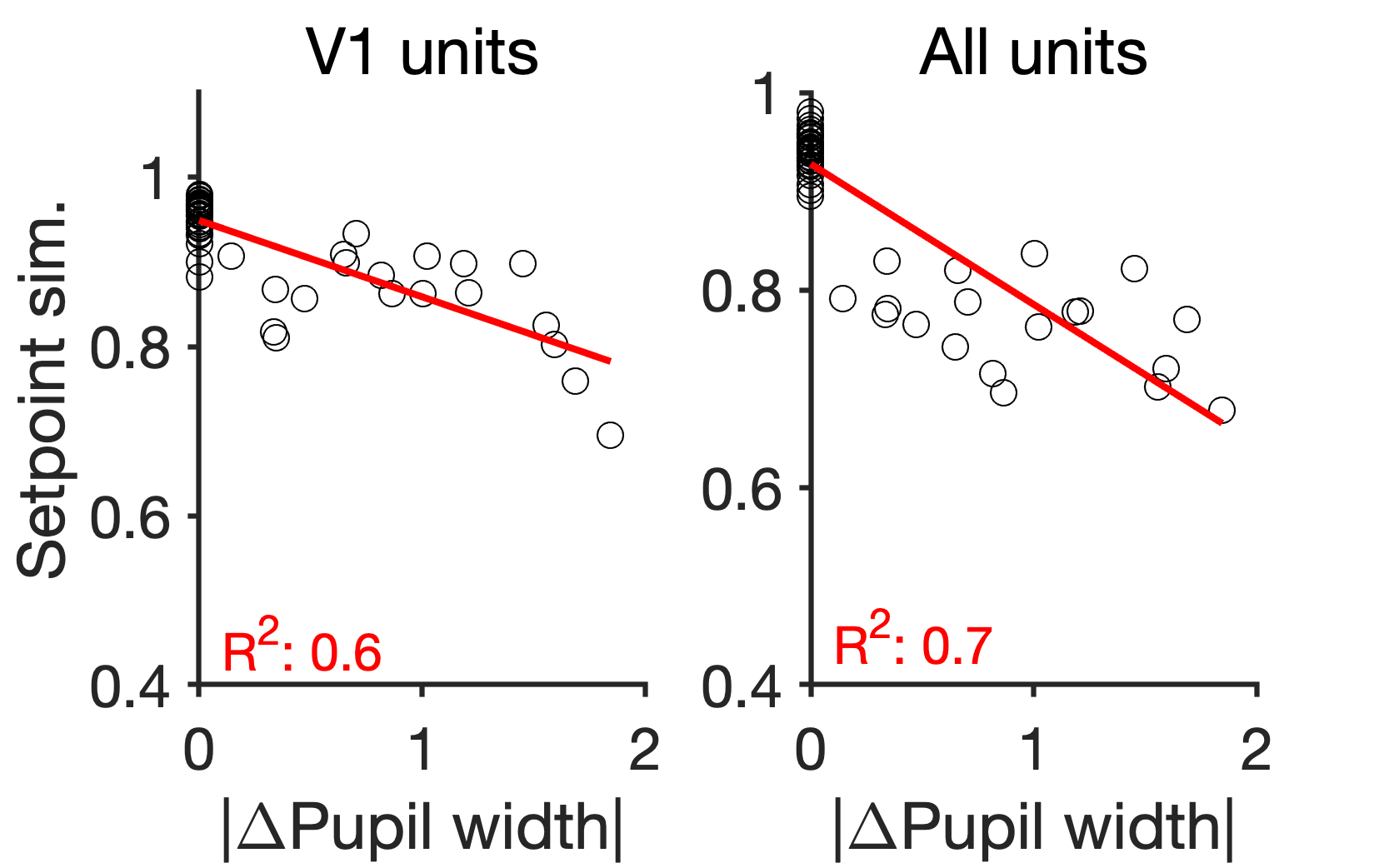

Supplement: Source code 1. [file elife-77907-code1.zip › Codes_eLife/Figure4/All/functional_connectivity_VISp_SetSim_ppd_all.png]

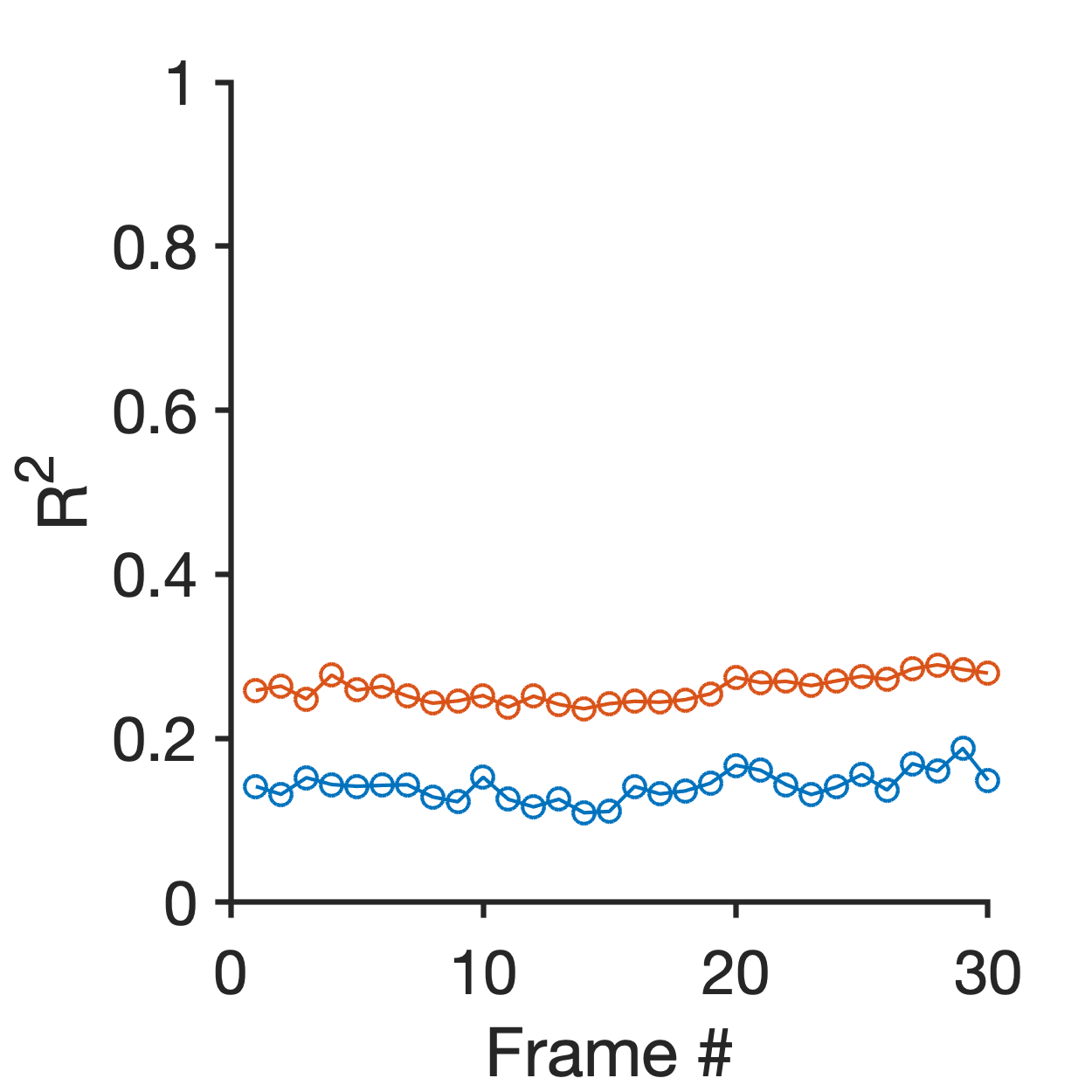

Supplement: Source code 1. [file elife-77907-code1.zip › Codes_eLife/Figure4/All/functional_connectivity_VISp_SetSim_ppd_stimWise.png]

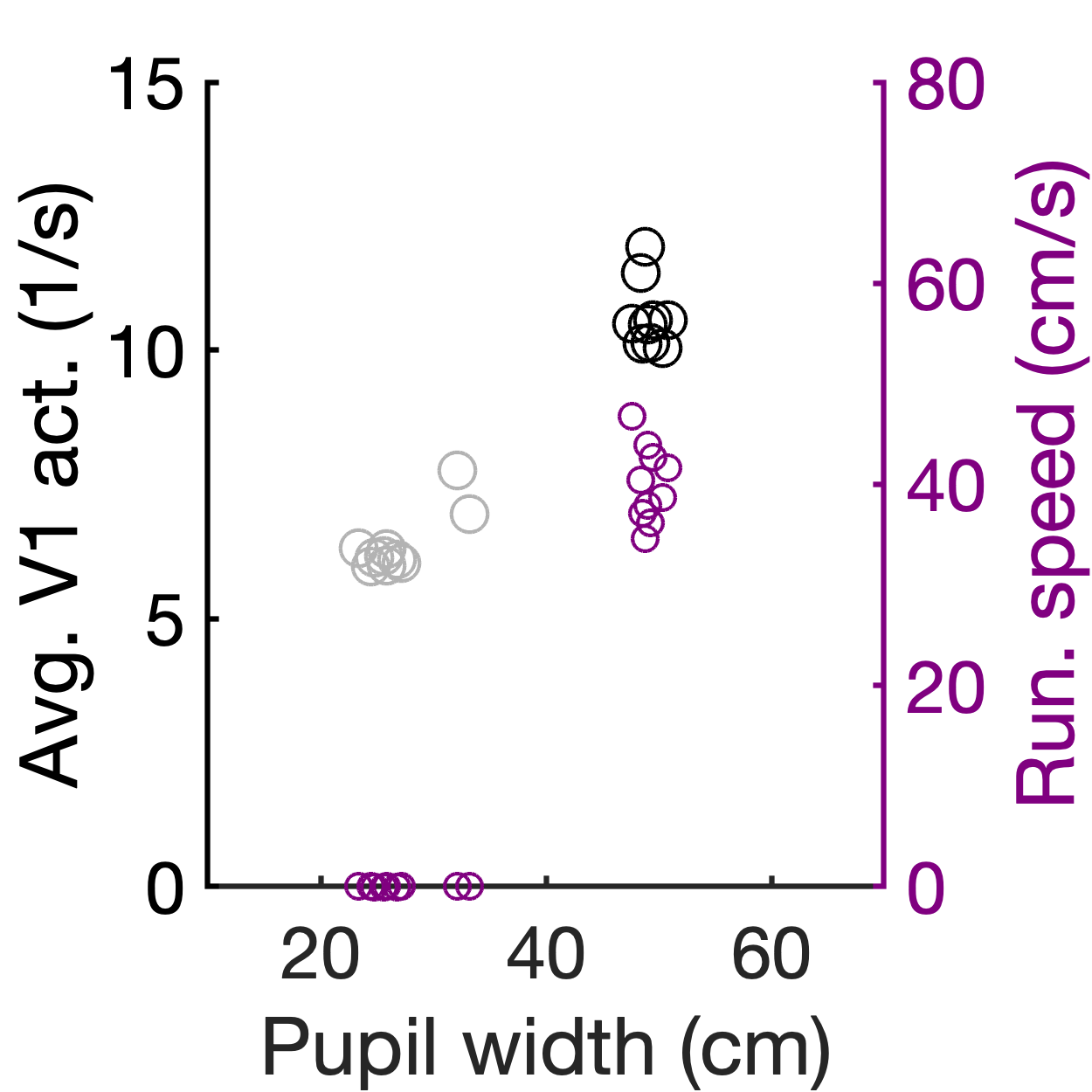

Supplement: Source code 1. [file elife-77907-code1.zip › Codes_eLife/Figure4/Examples/brain_observatory_1.1_VISp_pplRunAct.png]

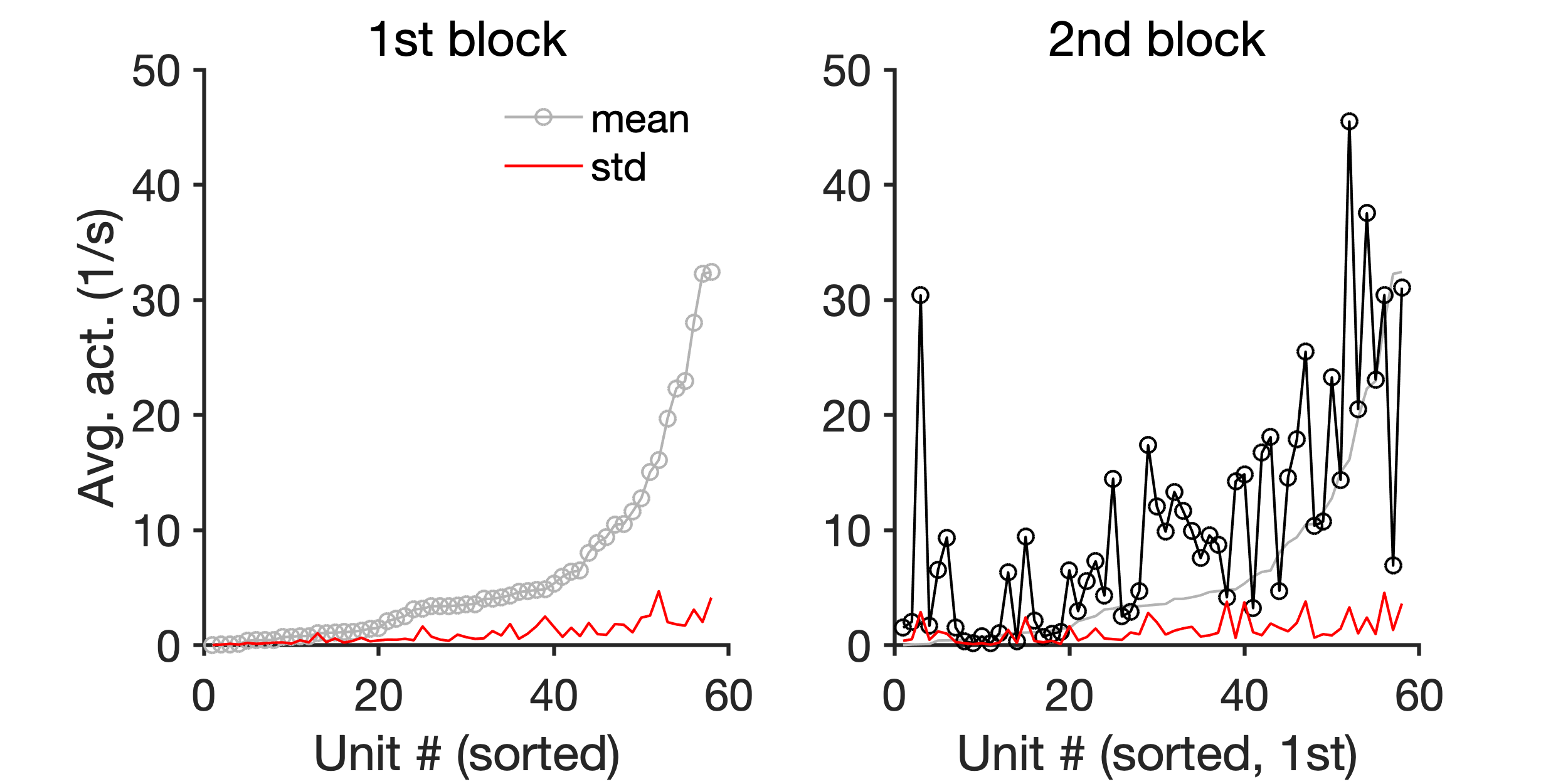

Supplement: Source code 1. [file elife-77907-code1.zip › Codes_eLife/Figure4/Examples/brain_observatory_1.1_VISp_ActAvgVar.png]

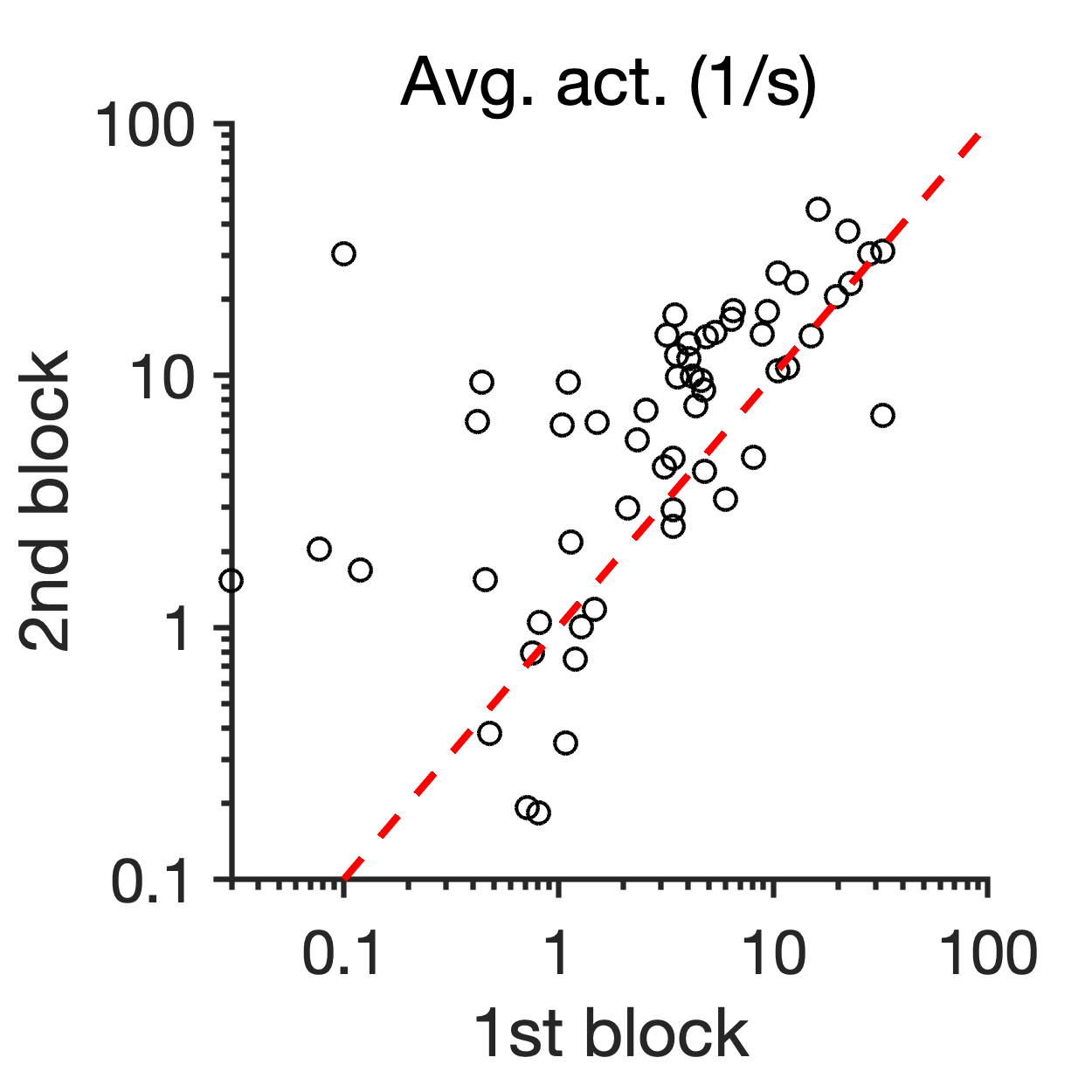

Supplement: Source code 1. [file elife-77907-code1.zip › Codes_eLife/Figure4/Examples/brain_observatory_1.1_VISp_ActAvg_2ndV1st.png]

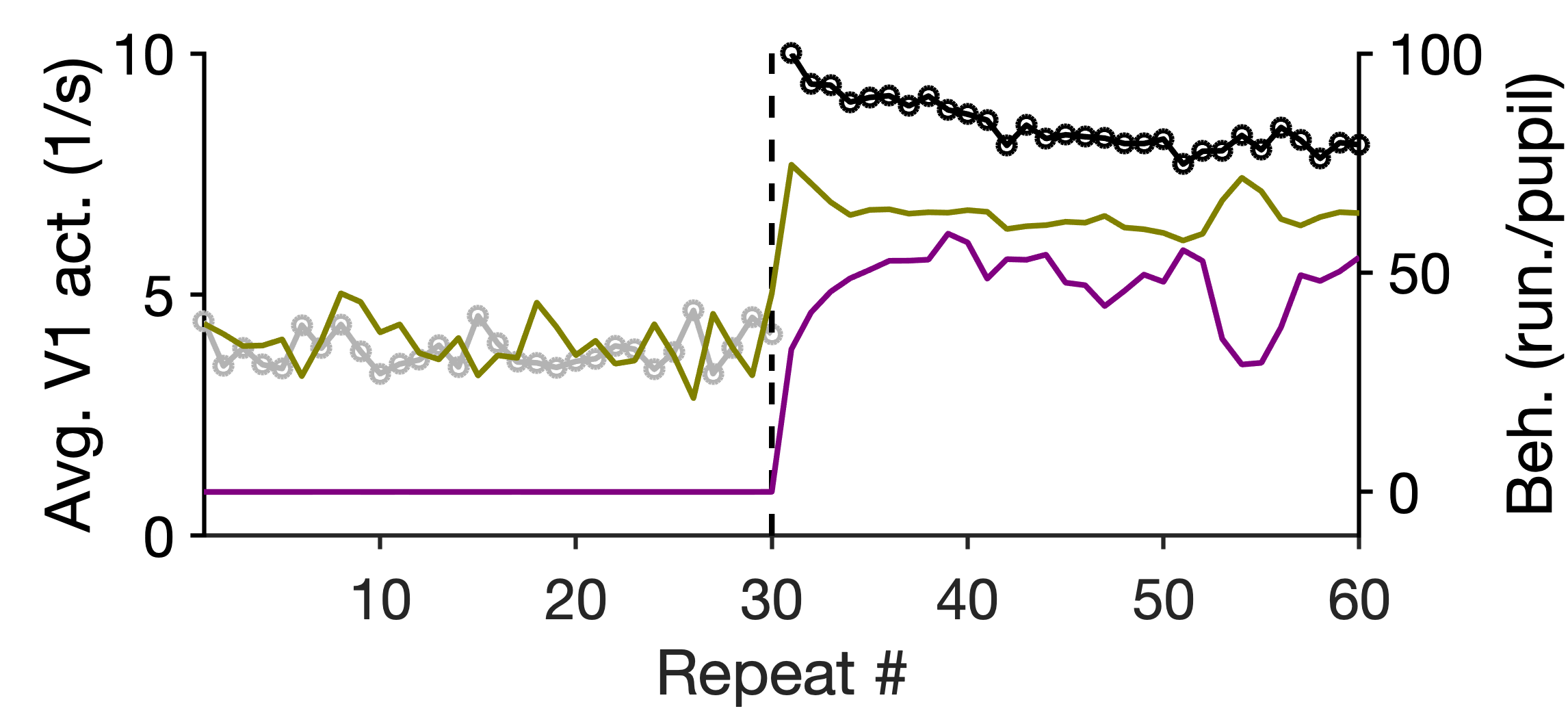

Supplement: Source code 1. [file elife-77907-code1.zip › Codes_eLife/Figure4/Examples/functional_connectivity_VISp_sampleAct.png]

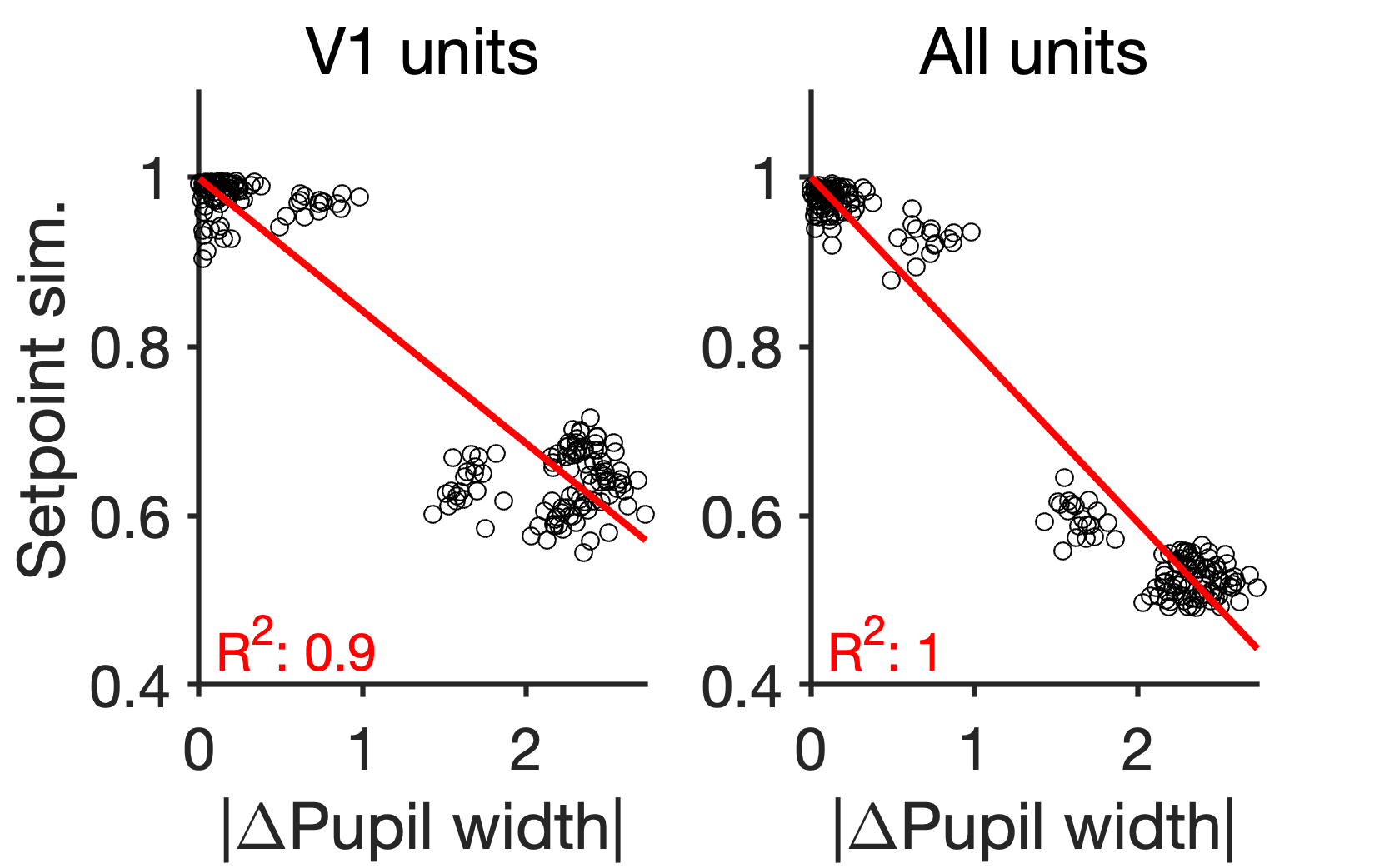

Supplement: Source code 1. [file elife-77907-code1.zip › Codes_eLife/Figure4/Examples/brain_observatory_1.1_VISp_SetSim_ppd.png]

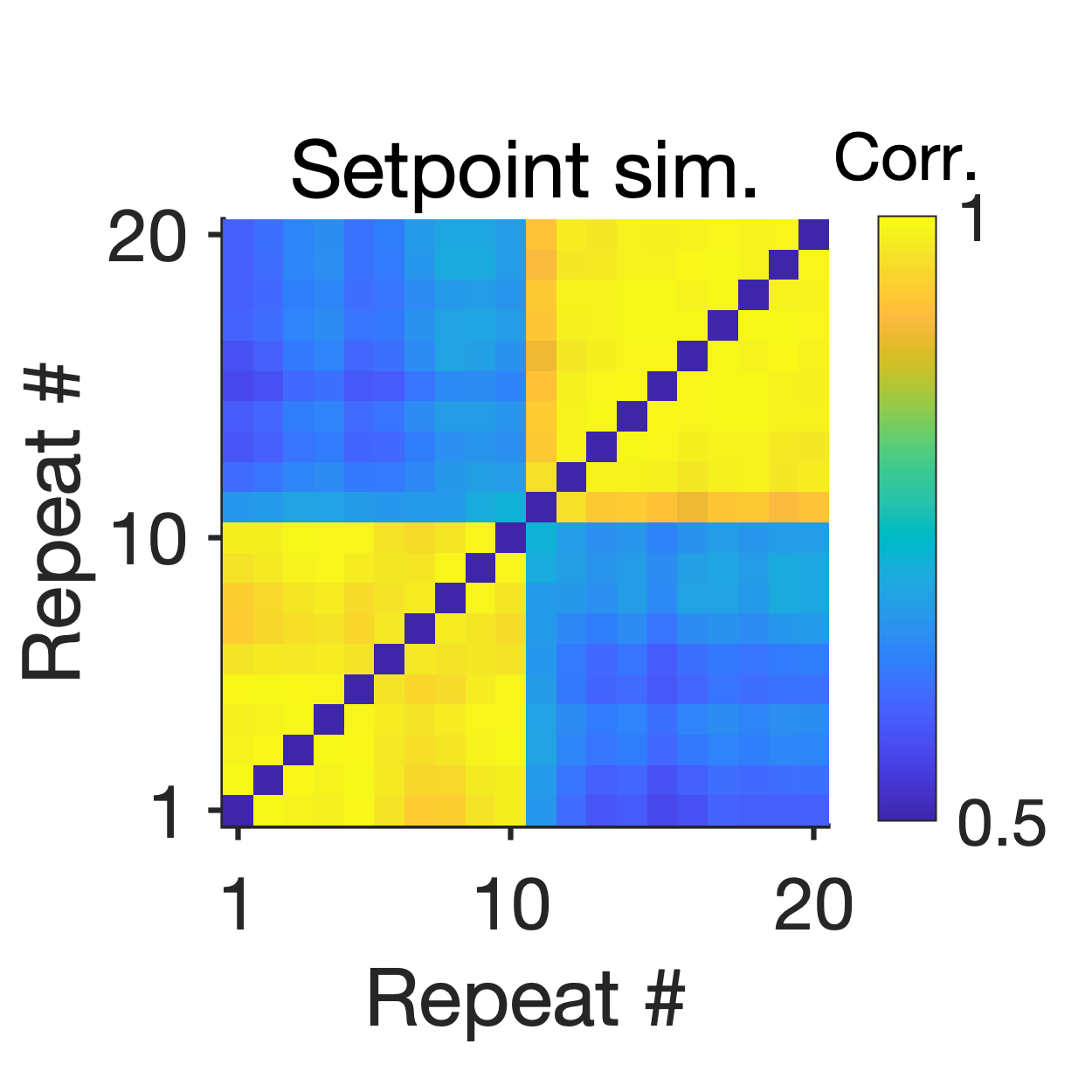

Supplement: Source code 1. [file elife-77907-code1.zip › Codes_eLife/Figure4/Examples/brain_observatory_1.1_VISp_SetSim.png]

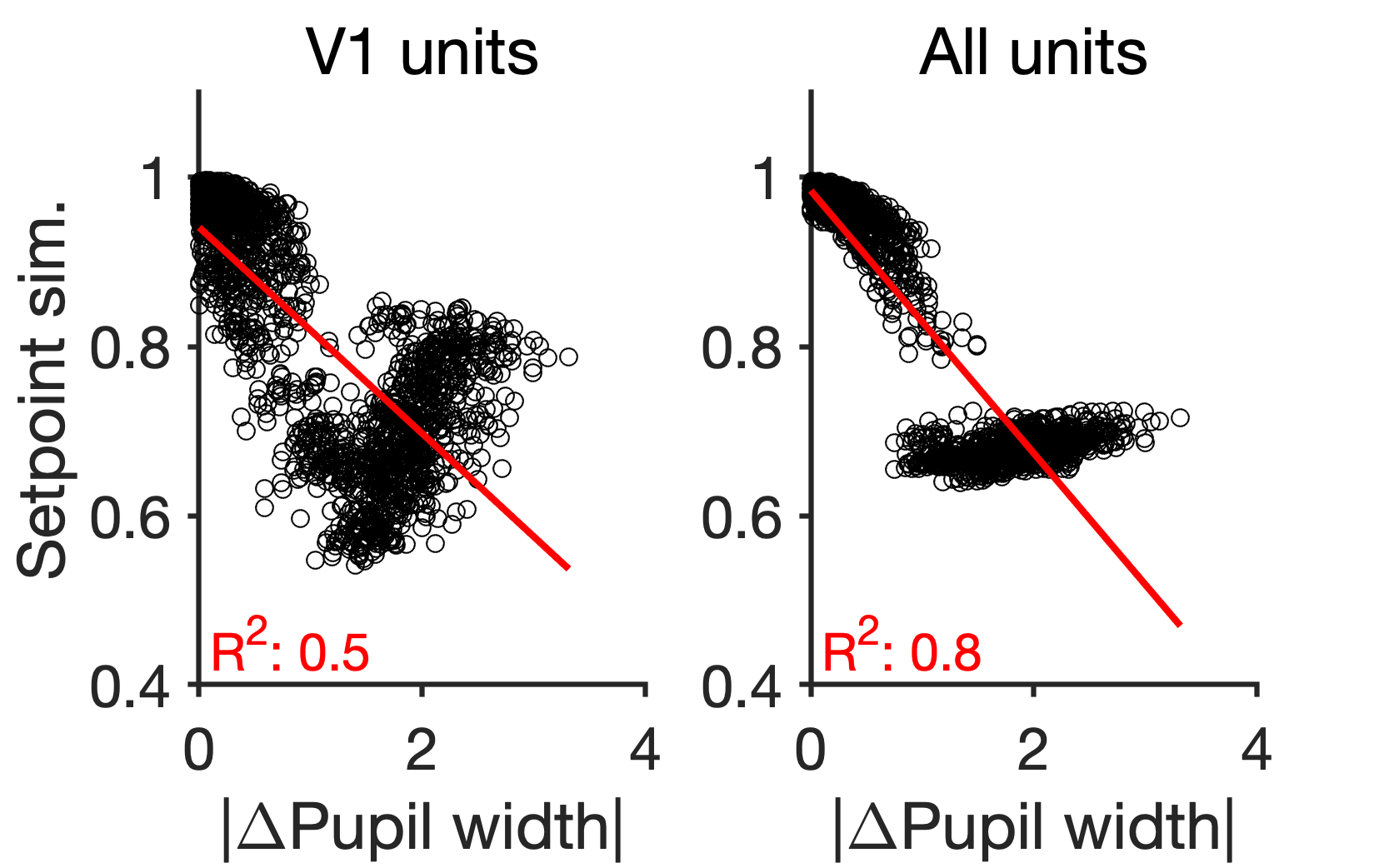

Supplement: Source code 1. [file elife-77907-code1.zip › Codes_eLife/Figure4/Examples/functional_connectivity_VISp_SetSim_ppd.png]

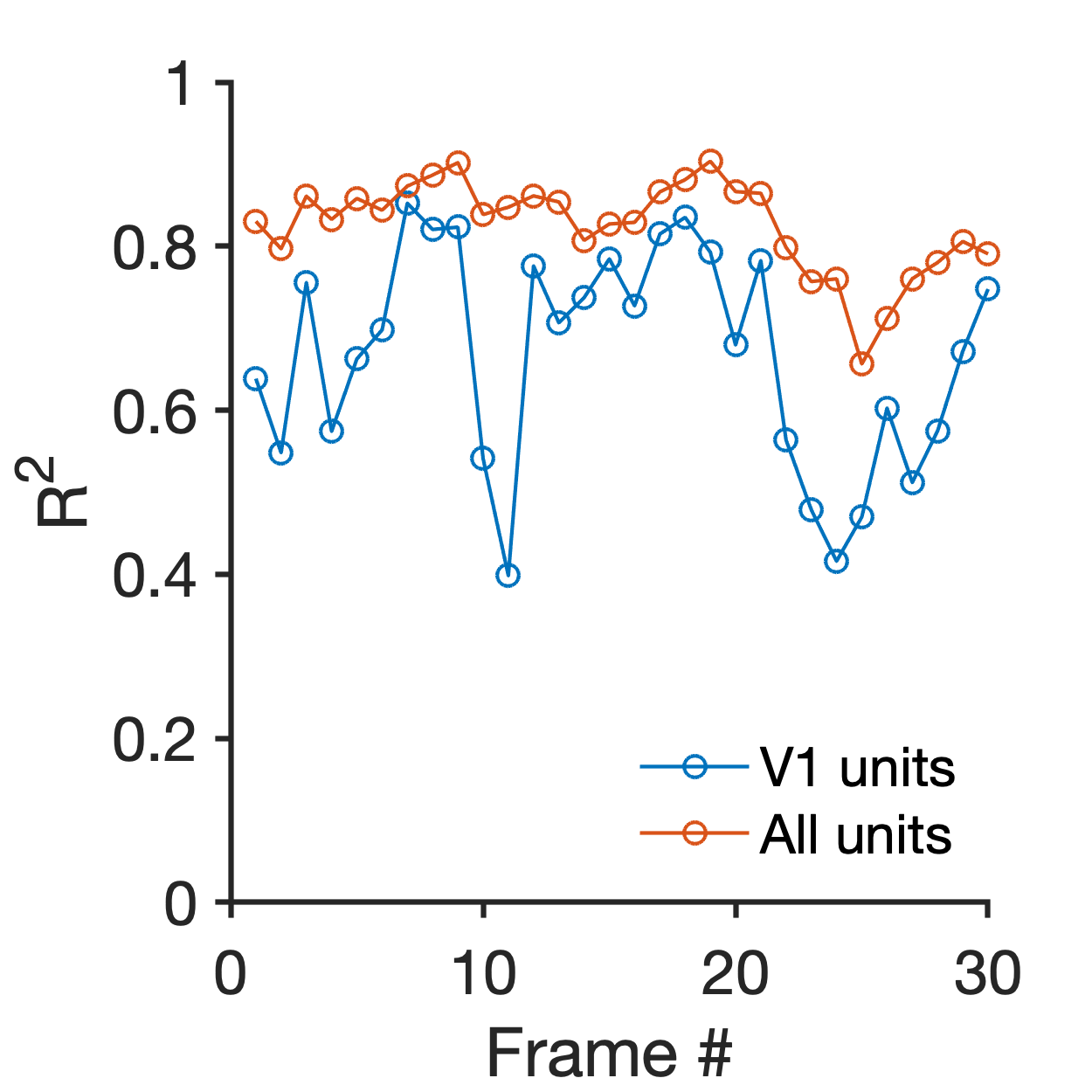

Supplement: Source code 1. [file elife-77907-code1.zip › Codes_eLife/Figure4/Examples/brain_observatory_1.1_VISp_SetSim_ppd_stimWise.png]

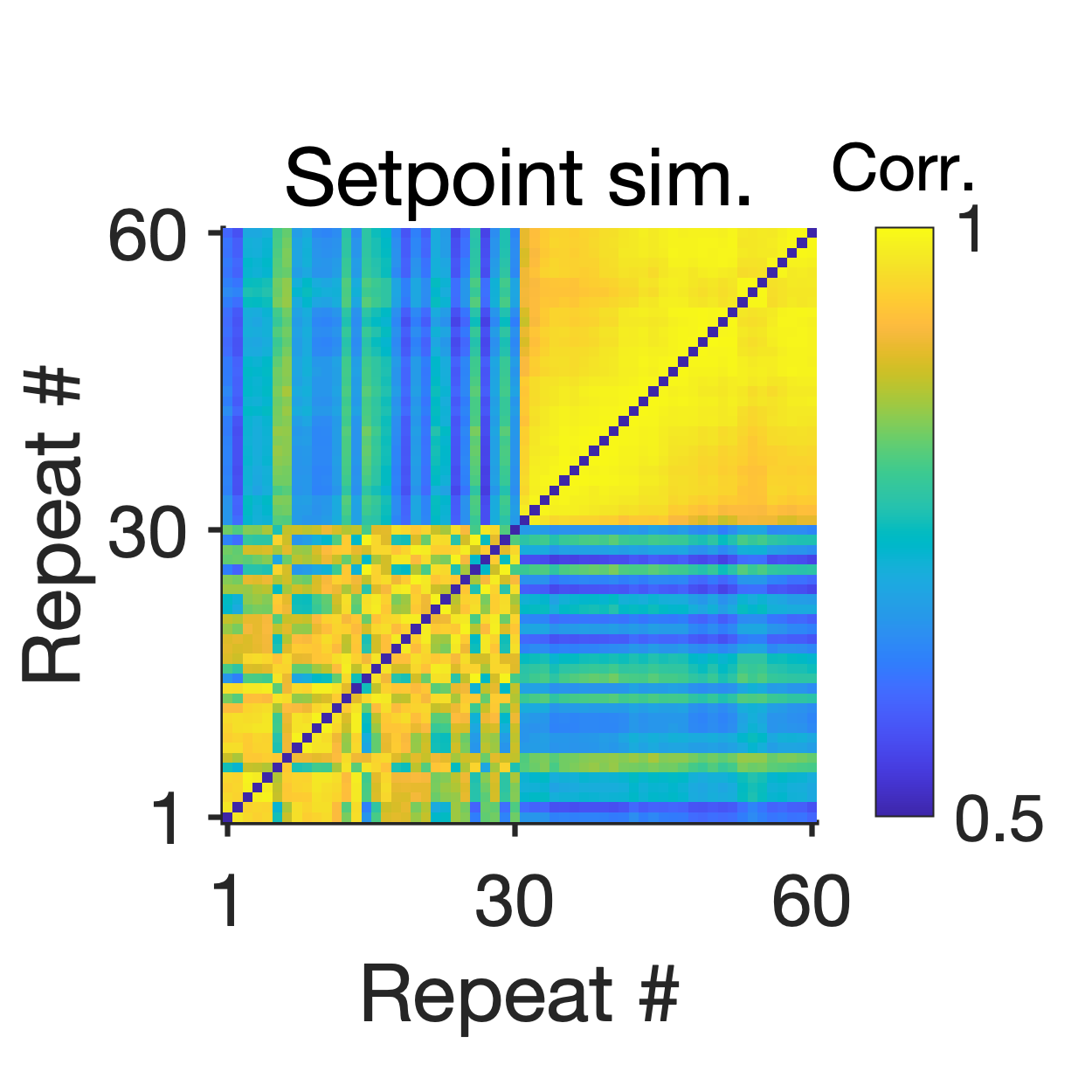

Supplement: Source code 1. [file elife-77907-code1.zip › Codes_eLife/Figure4/Examples/functional_connectivity_VISp_SetSim.png]

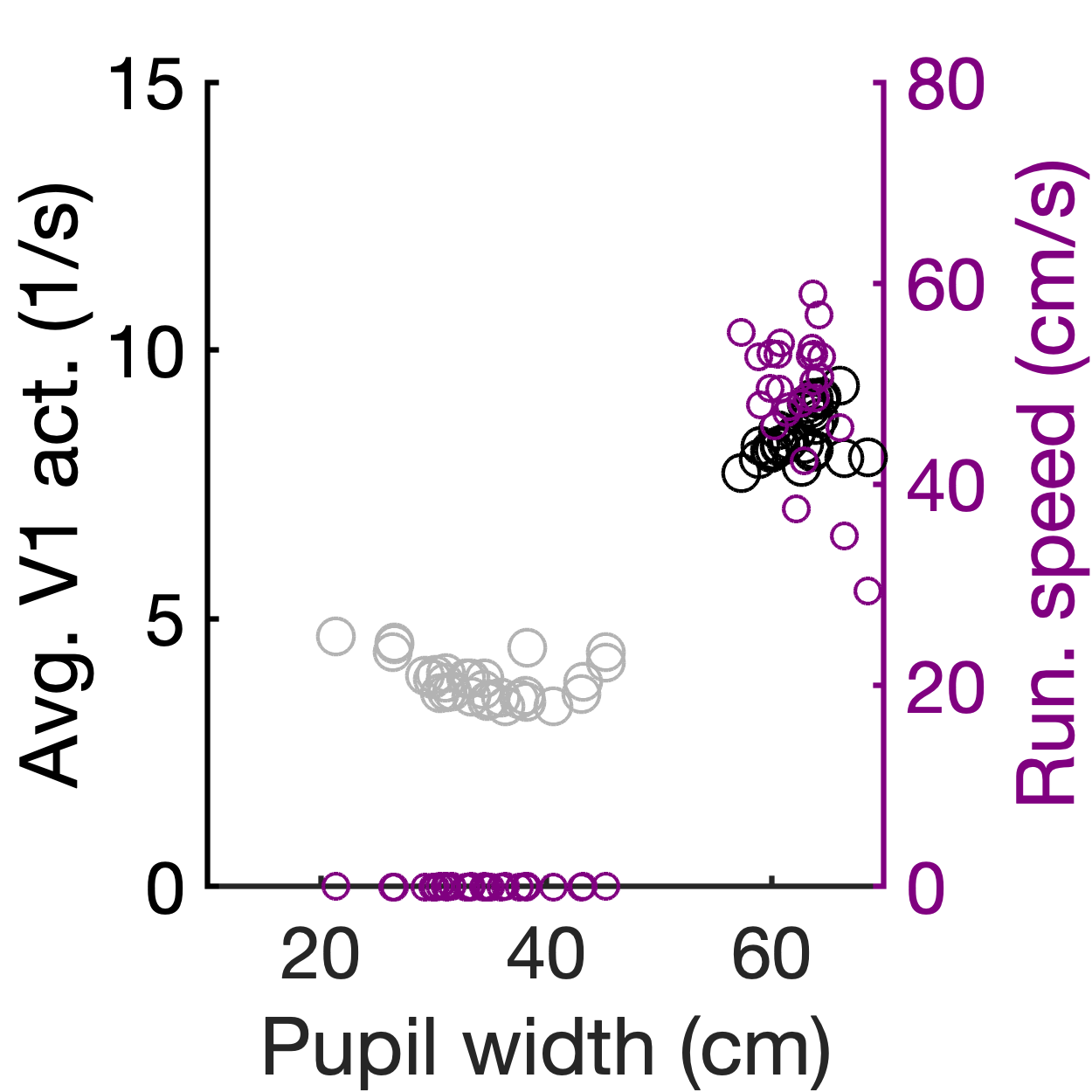

Supplement: Source code 1. [file elife-77907-code1.zip › Codes_eLife/Figure4/Examples/functional_connectivity_VISp_pplRunAct.png]

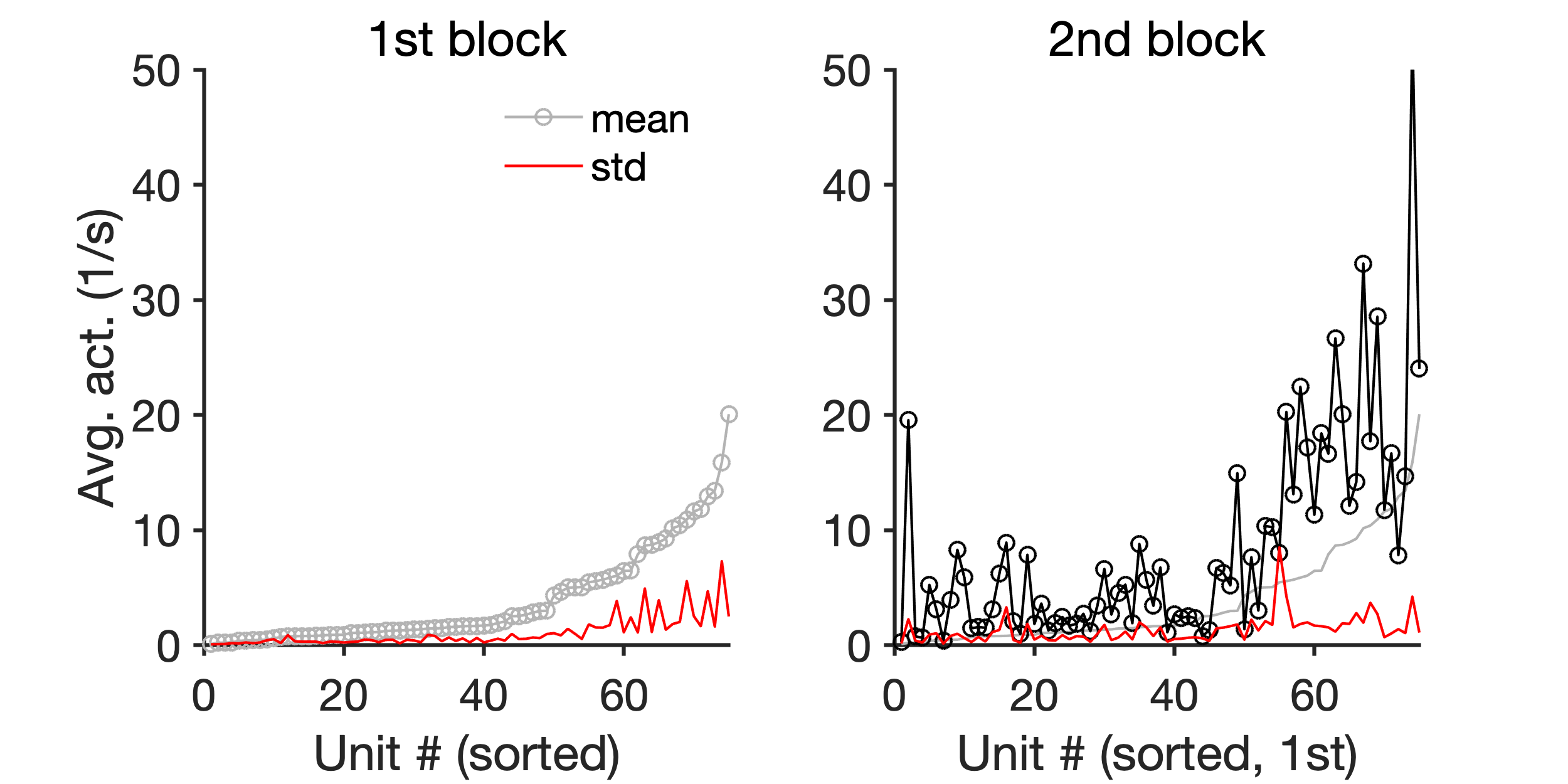

Supplement: Source code 1. [file elife-77907-code1.zip › Codes_eLife/Figure4/Examples/functional_connectivity_VISp_ActAvgVar.png]

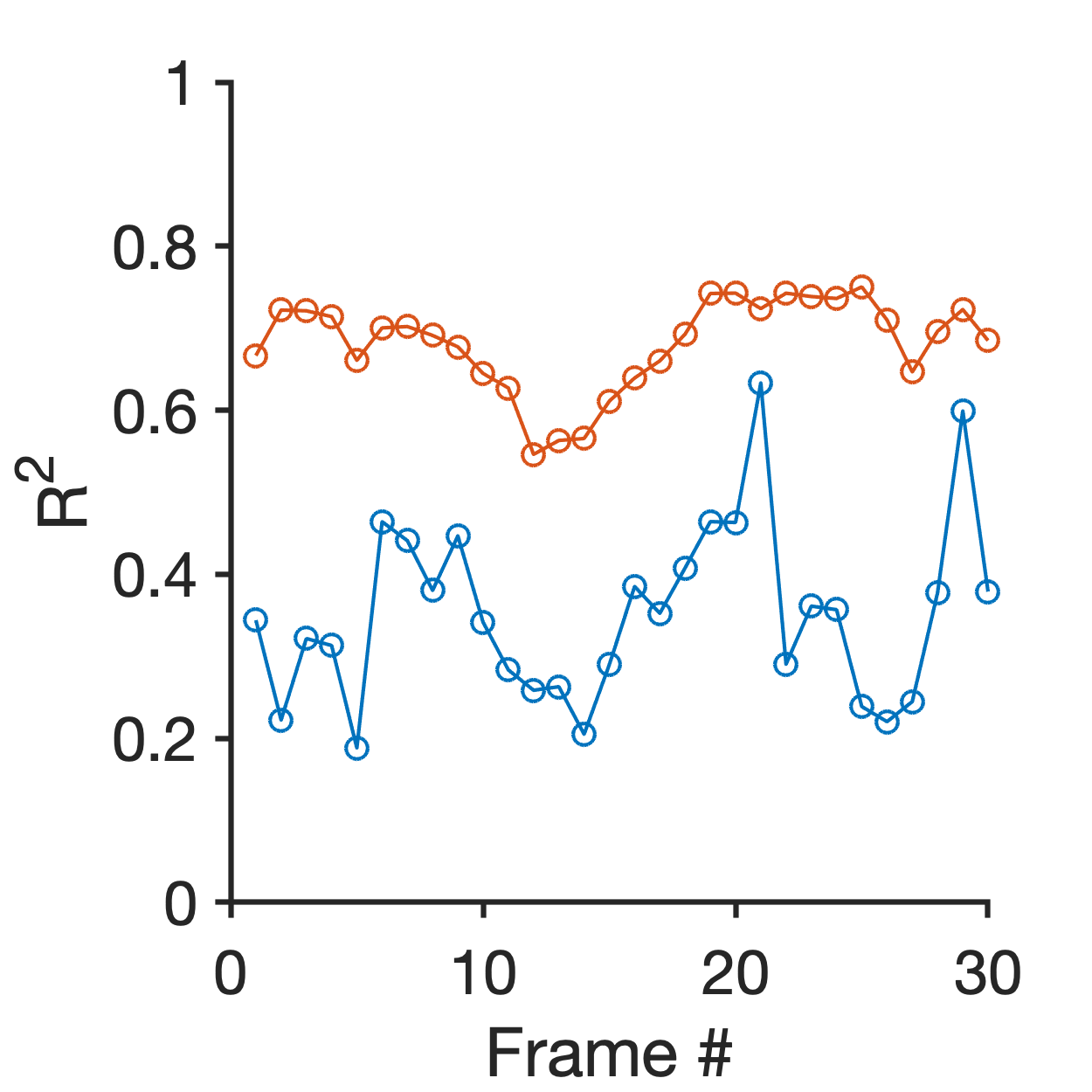

Supplement: Source code 1. [file elife-77907-code1.zip › Codes_eLife/Figure4/Examples/functional_connectivity_VISp_SetSim_ppd_stimWise.png]

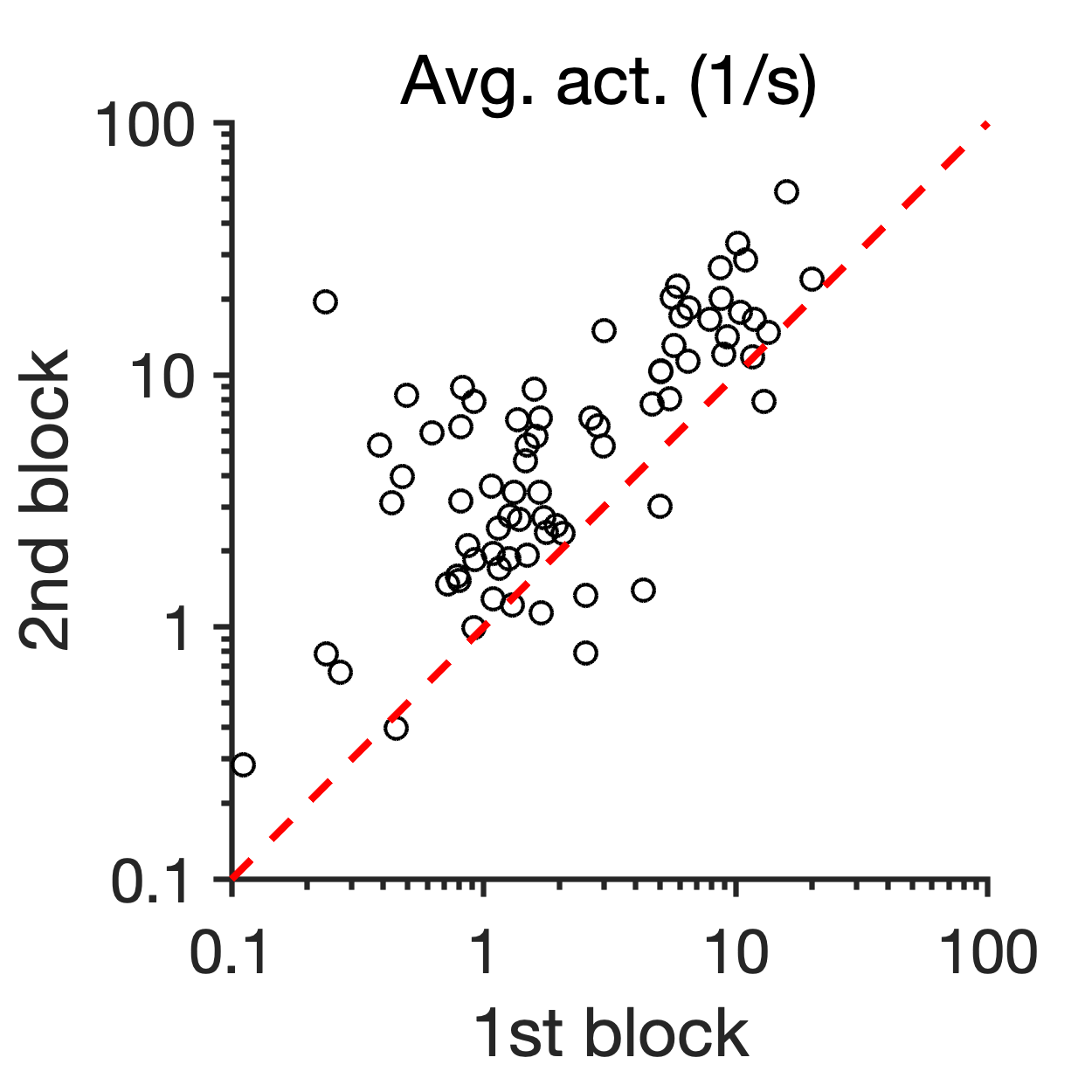

Supplement: Source code 1. [file elife-77907-code1.zip › Codes_eLife/Figure4/Examples/functional_connectivity_VISp_ActAvg_2ndV1st.png]

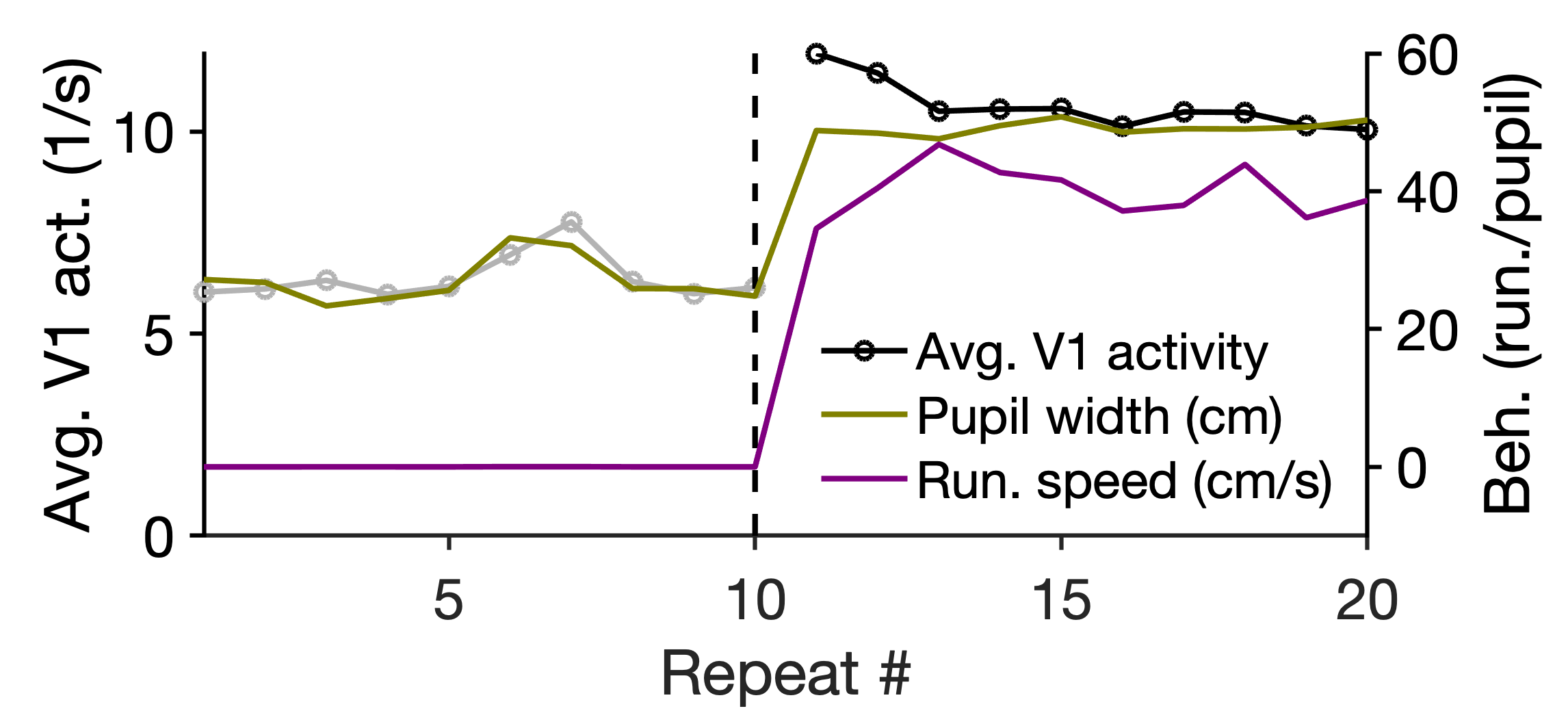

Supplement: Source code 1. [file elife-77907-code1.zip › Codes_eLife/Figure4/Examples/brain_observatory_1.1_VISp_sampleAct.png]

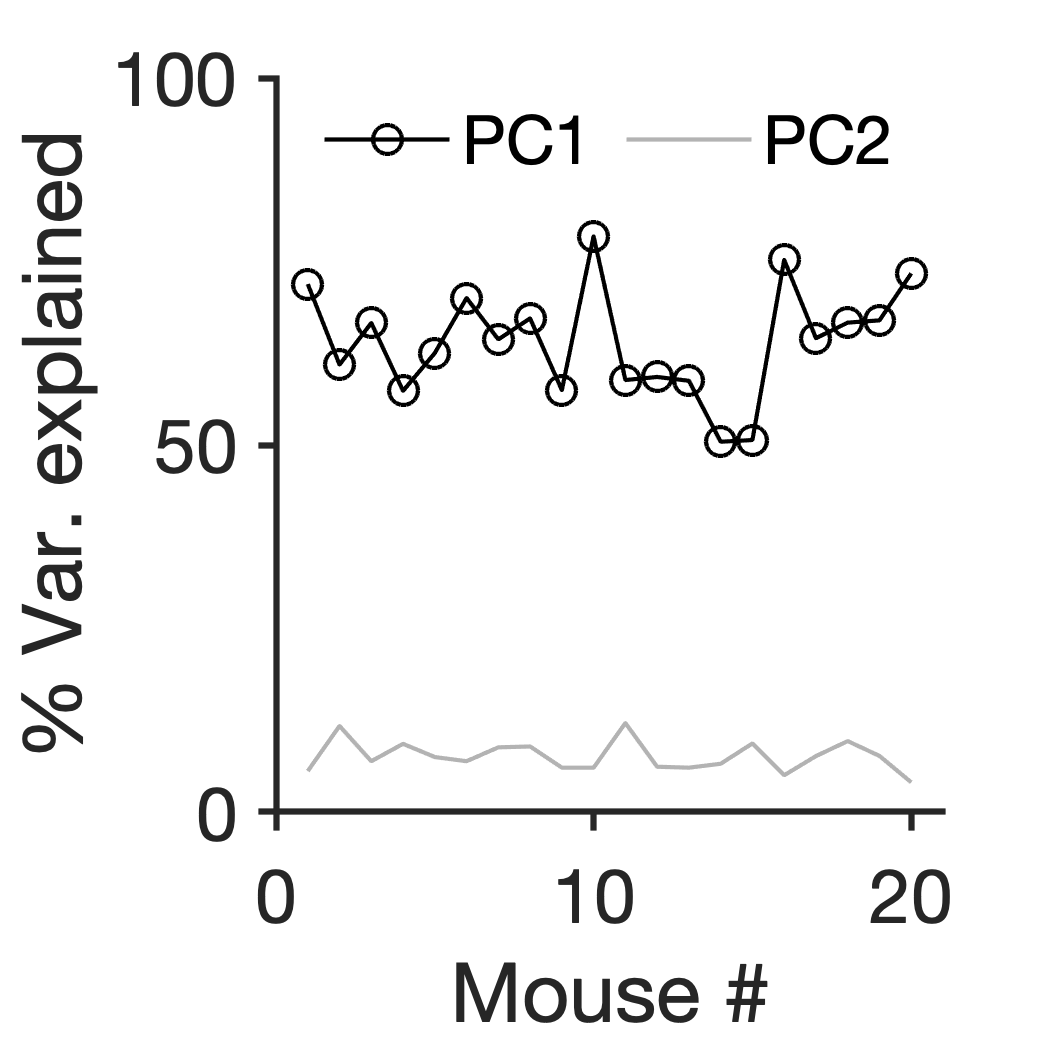

Supplement: Source code 1. [file elife-77907-code1.zip › Codes_eLife/Figure3/all/brain_observatory_1.1/pc_space_VISp__13.png]

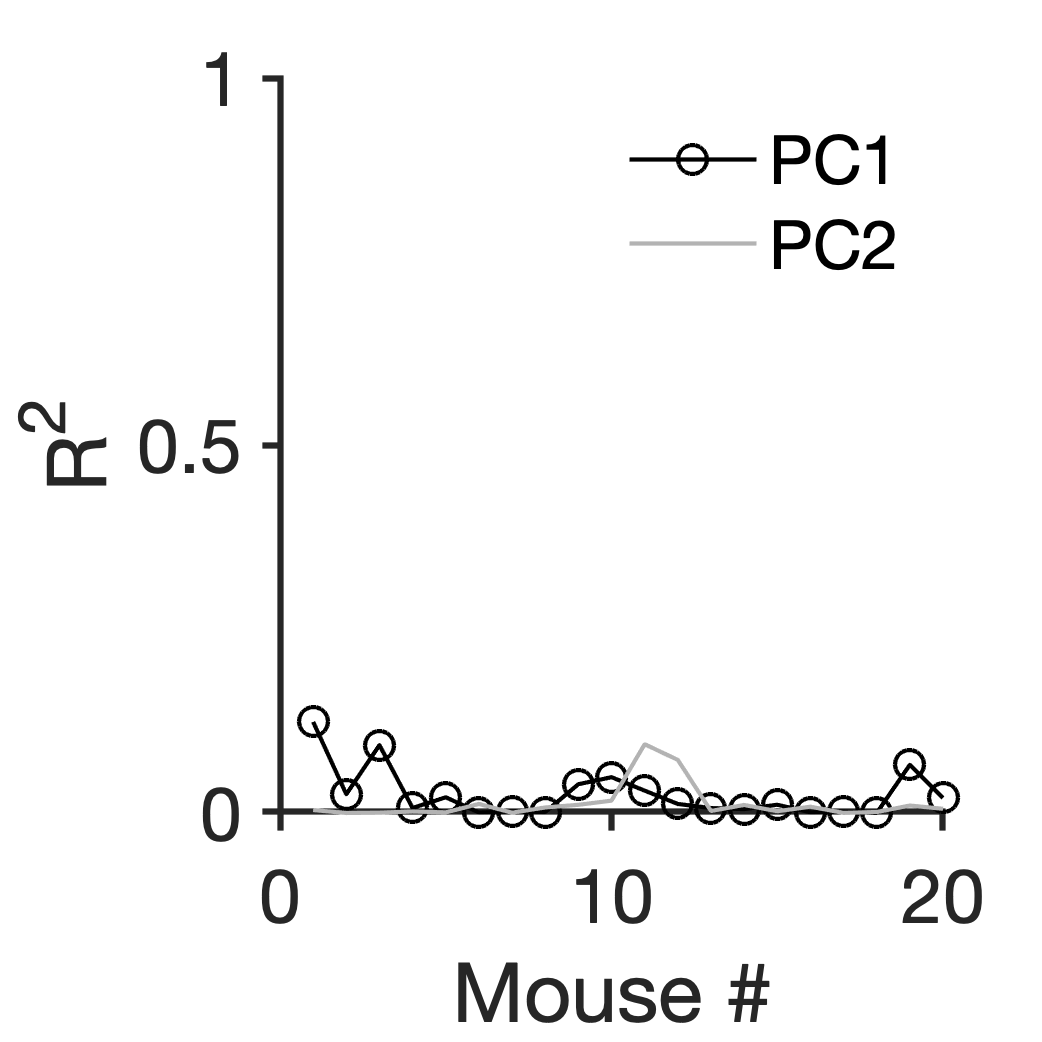

Supplement: Source code 1. [file elife-77907-code1.zip › Codes_eLife/Figure3/all/brain_observatory_1.1/pc_space_VISp__12.png]

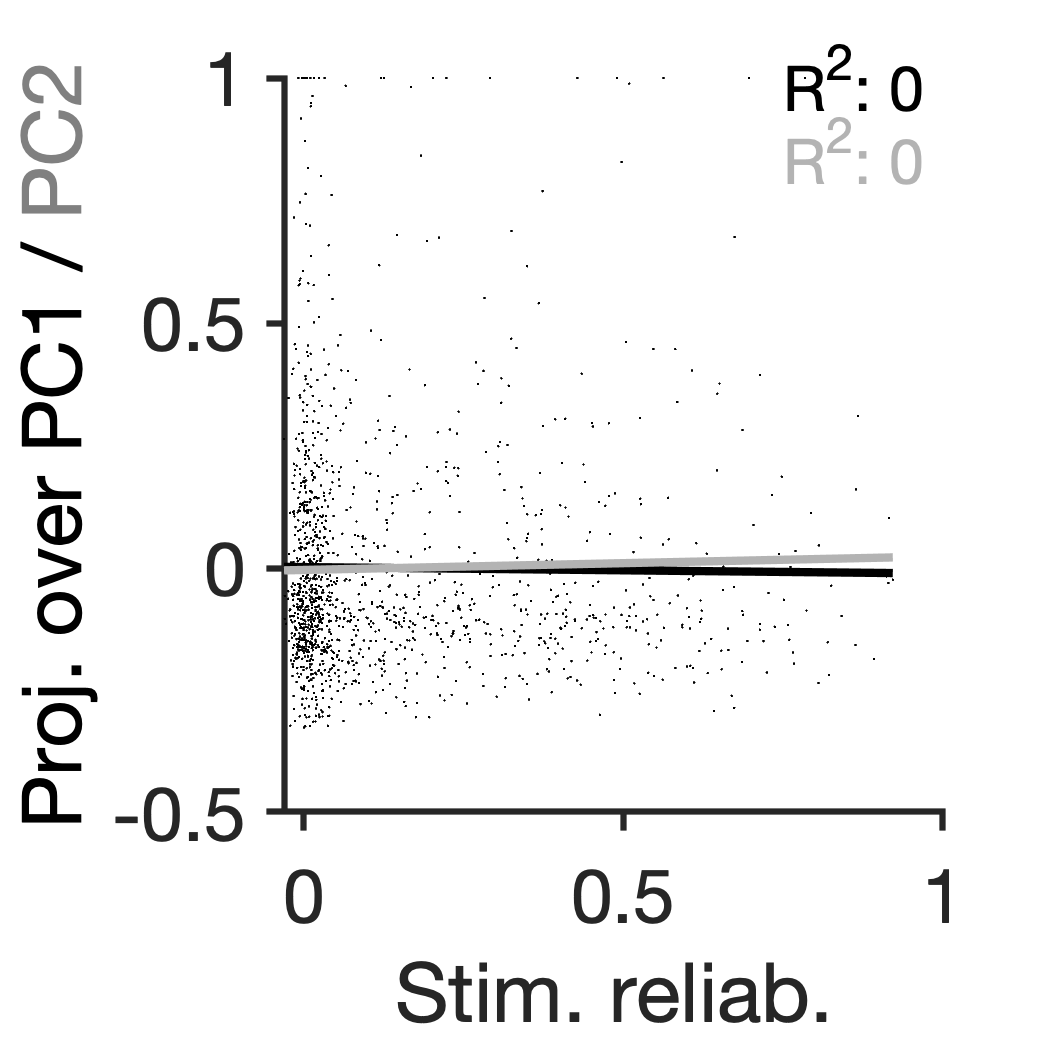

Supplement: Source code 1. [file elife-77907-code1.zip › Codes_eLife/Figure3/all/brain_observatory_1.1/pc_space_VISp__11.png]

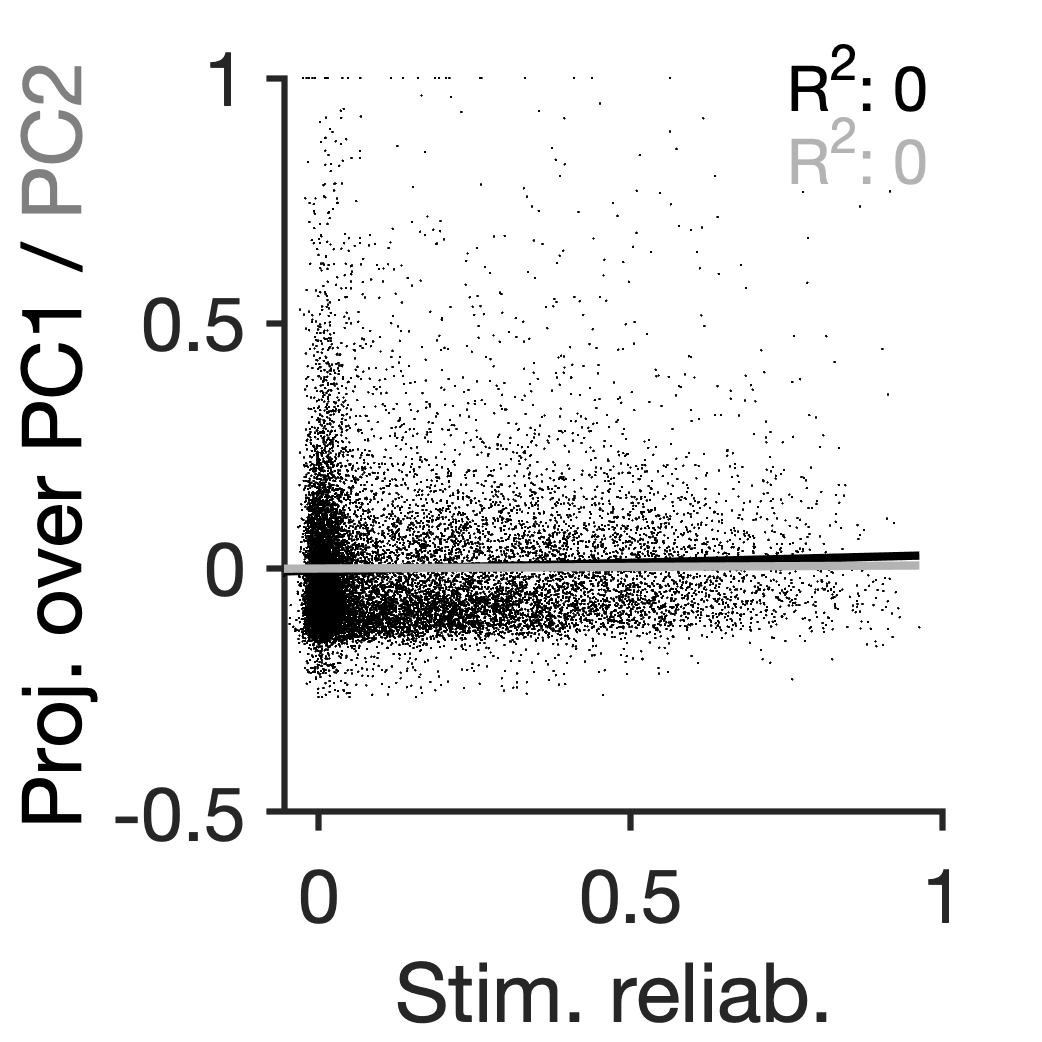

Supplement: Source code 1. [file elife-77907-code1.zip › Codes_eLife/Figure3/all/brain_observatory_1.1/pc_space_all__11.png]

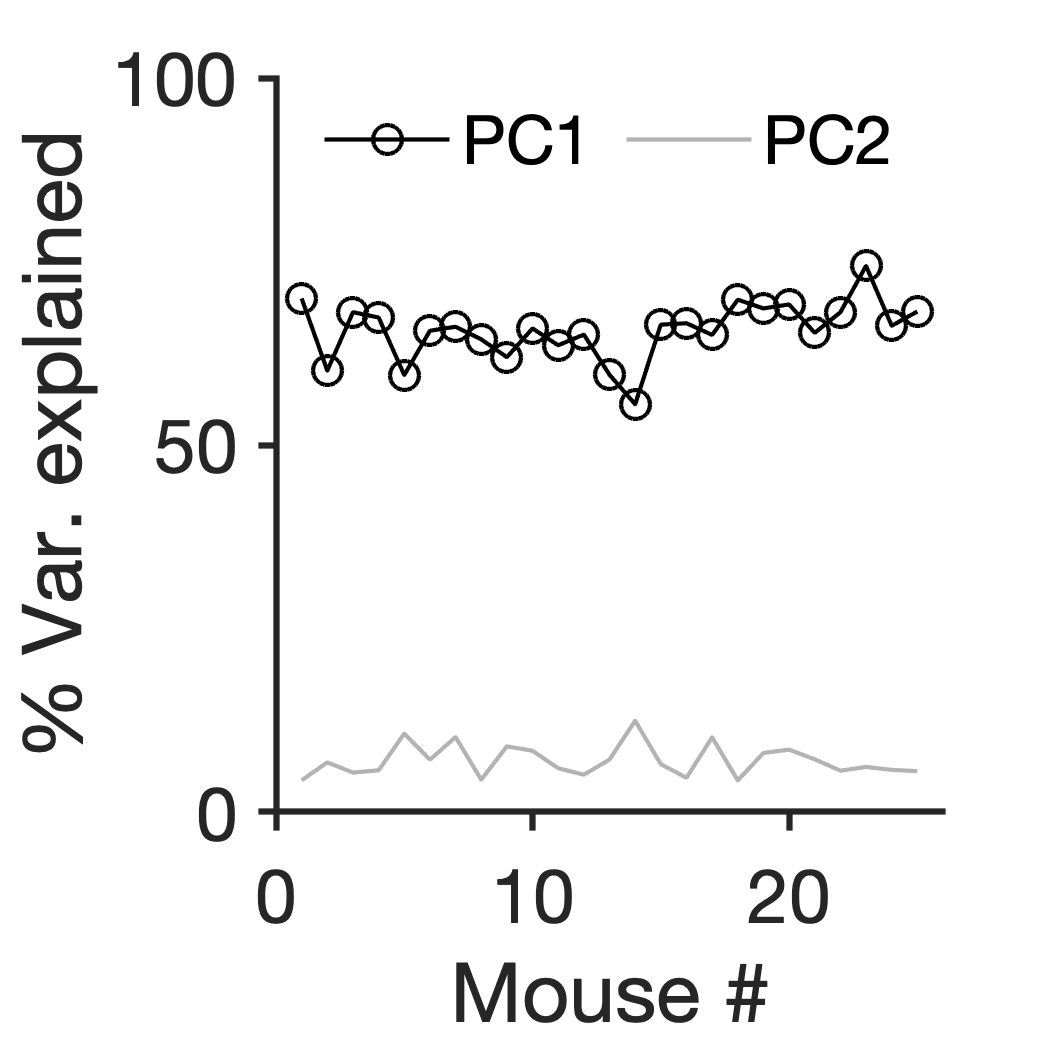

Supplement: Source code 1. [file elife-77907-code1.zip › Codes_eLife/Figure3/all/brain_observatory_1.1/pc_space_all__13.png]

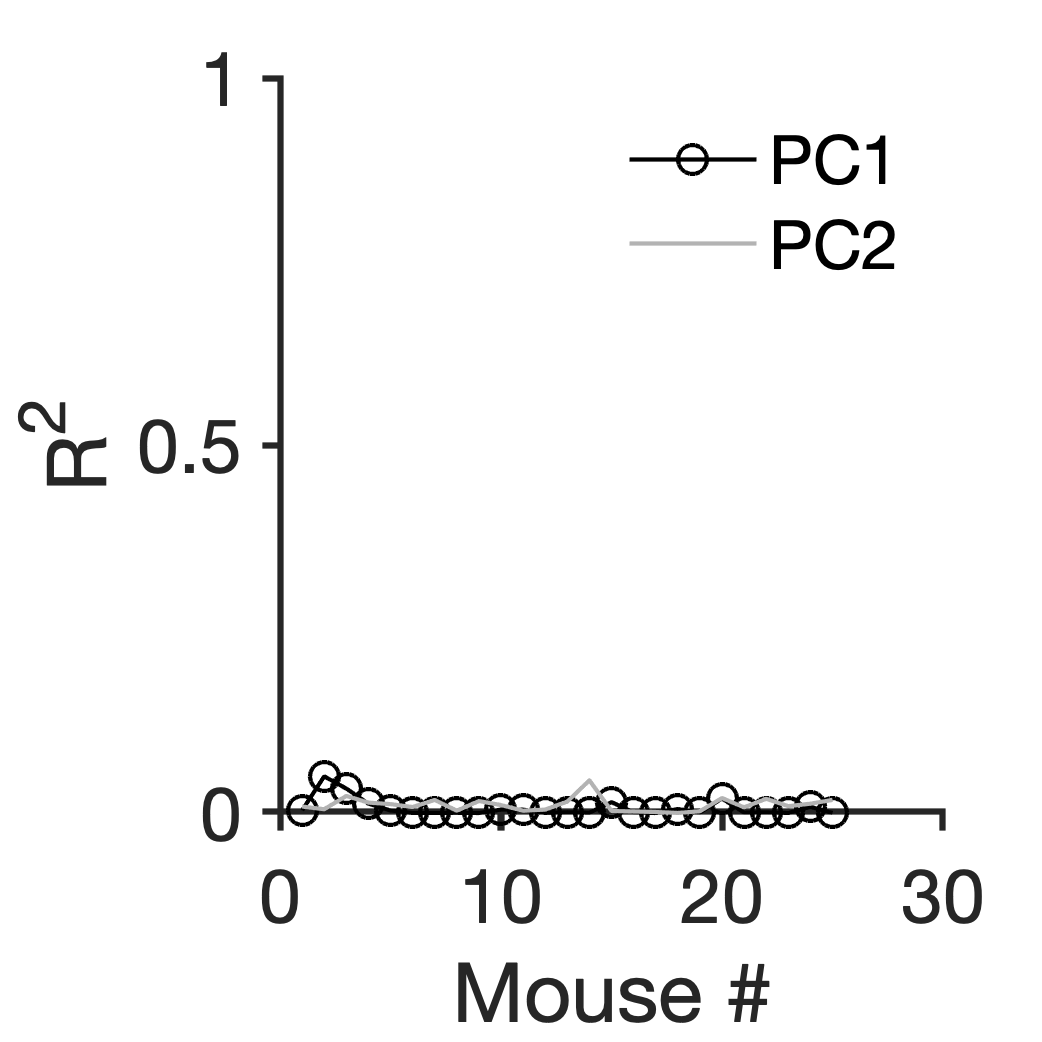

Supplement: Source code 1. [file elife-77907-code1.zip › Codes_eLife/Figure3/all/brain_observatory_1.1/pc_space_all__12.png]

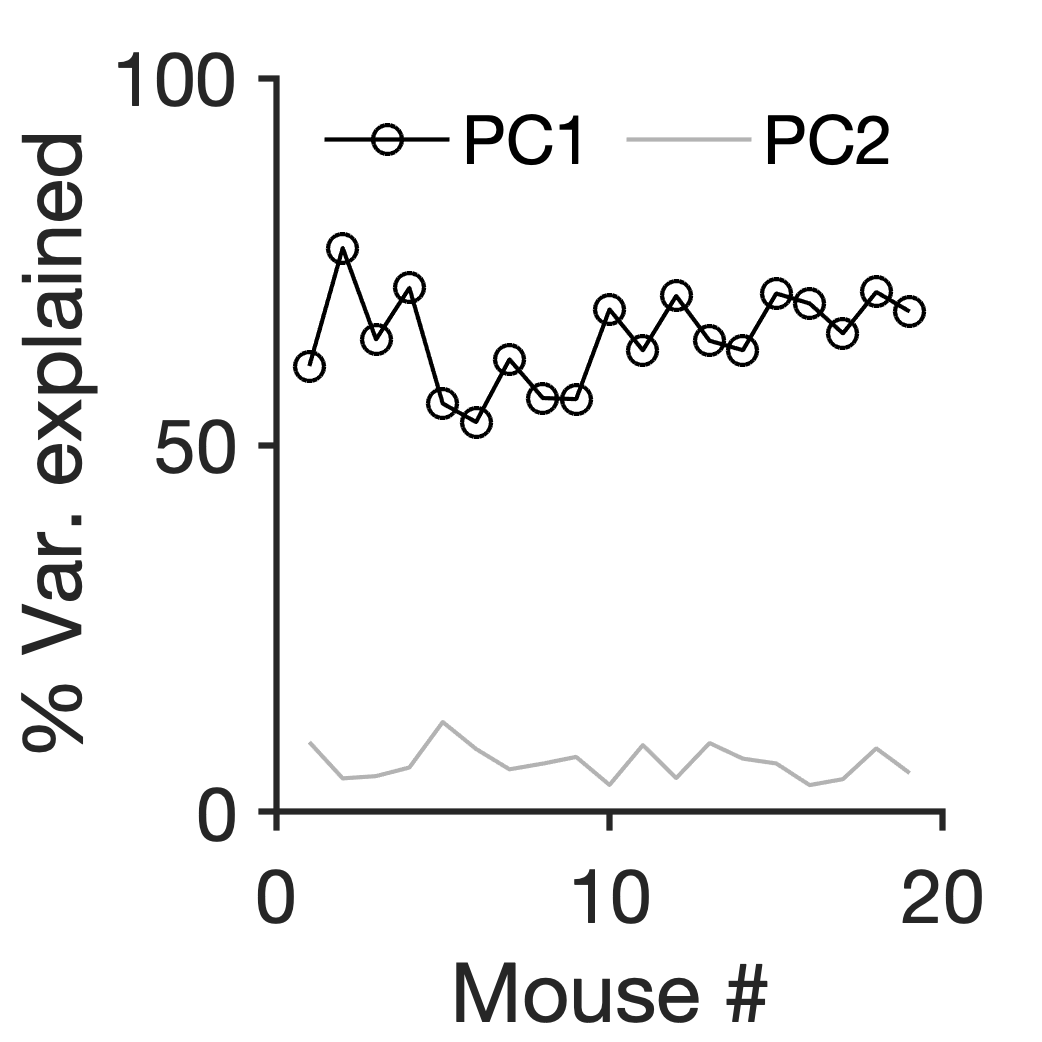

Supplement: Source code 1. [file elife-77907-code1.zip › Codes_eLife/Figure3/all/functional_connectivity/pc_space_VISp__13.png]

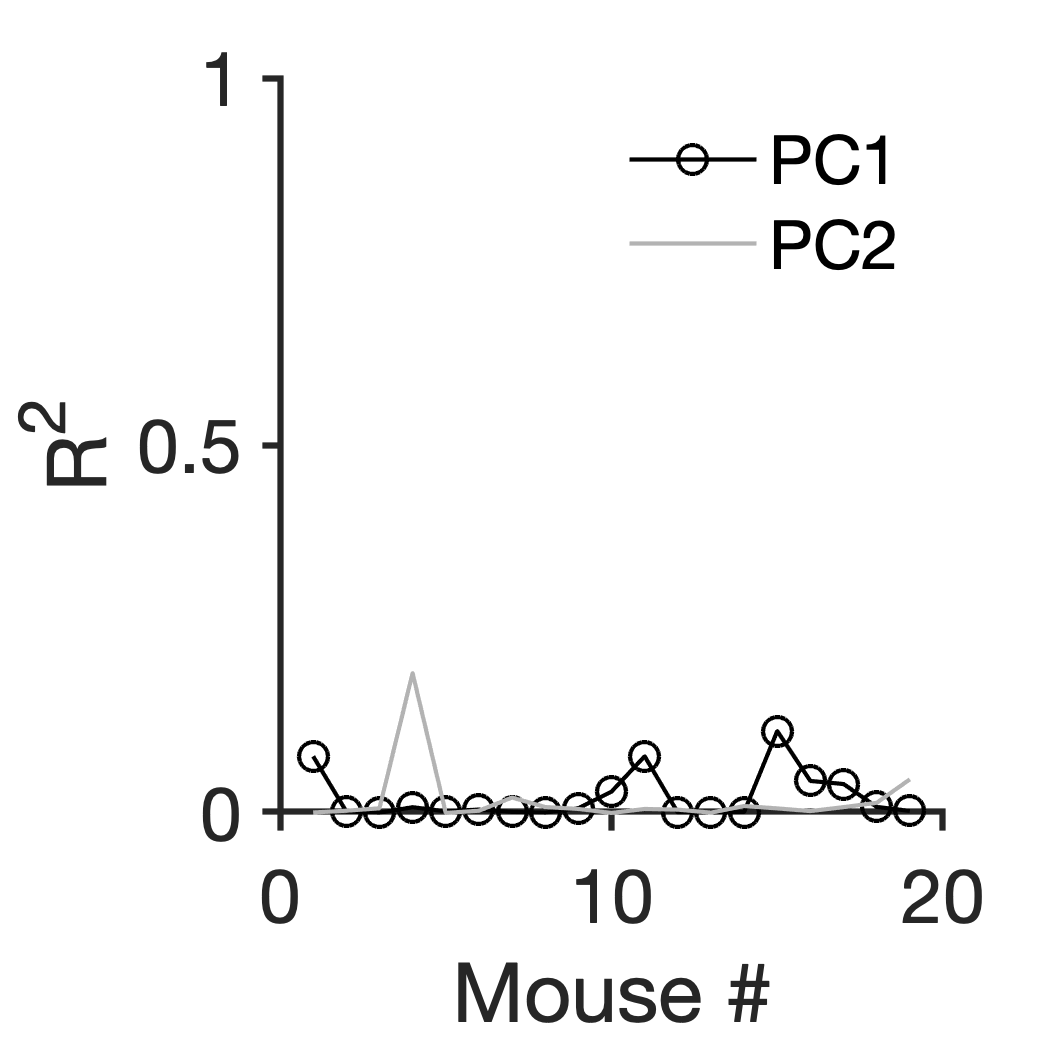

Supplement: Source code 1. [file elife-77907-code1.zip › Codes_eLife/Figure3/all/functional_connectivity/pc_space_VISp__12.png]

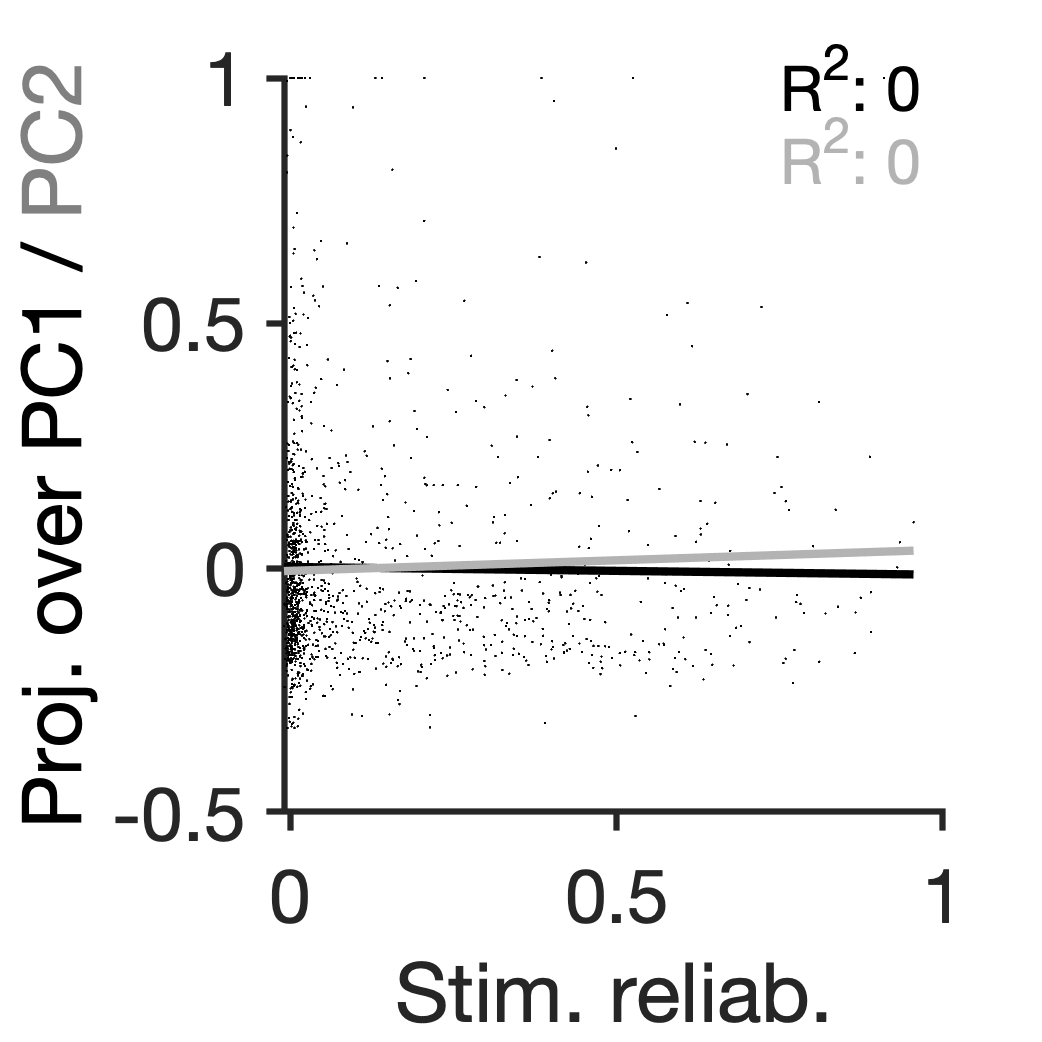

Supplement: Source code 1. [file elife-77907-code1.zip › Codes_eLife/Figure3/all/functional_connectivity/pc_space_VISp__11.png]

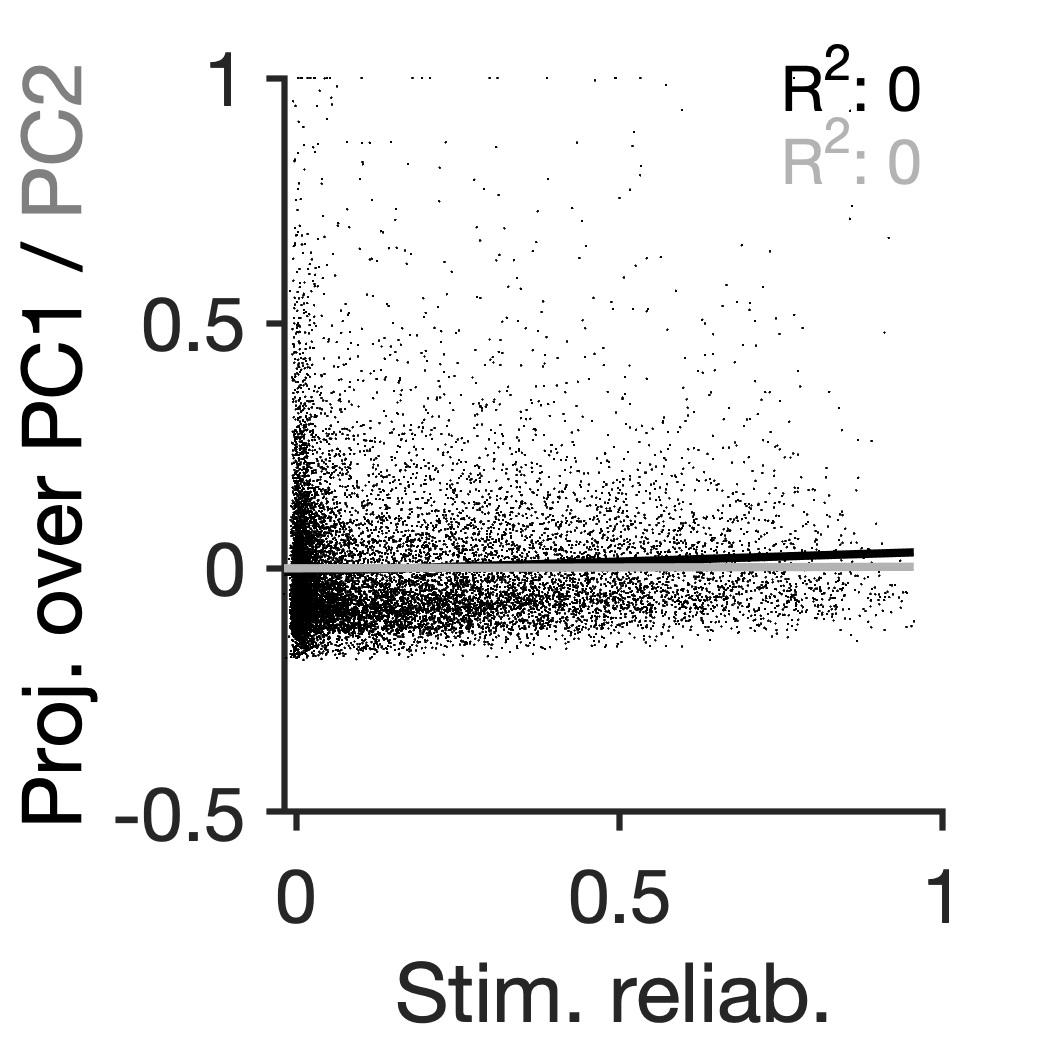

Supplement: Source code 1. [file elife-77907-code1.zip › Codes_eLife/Figure3/all/functional_connectivity/pc_space_all__11.png]

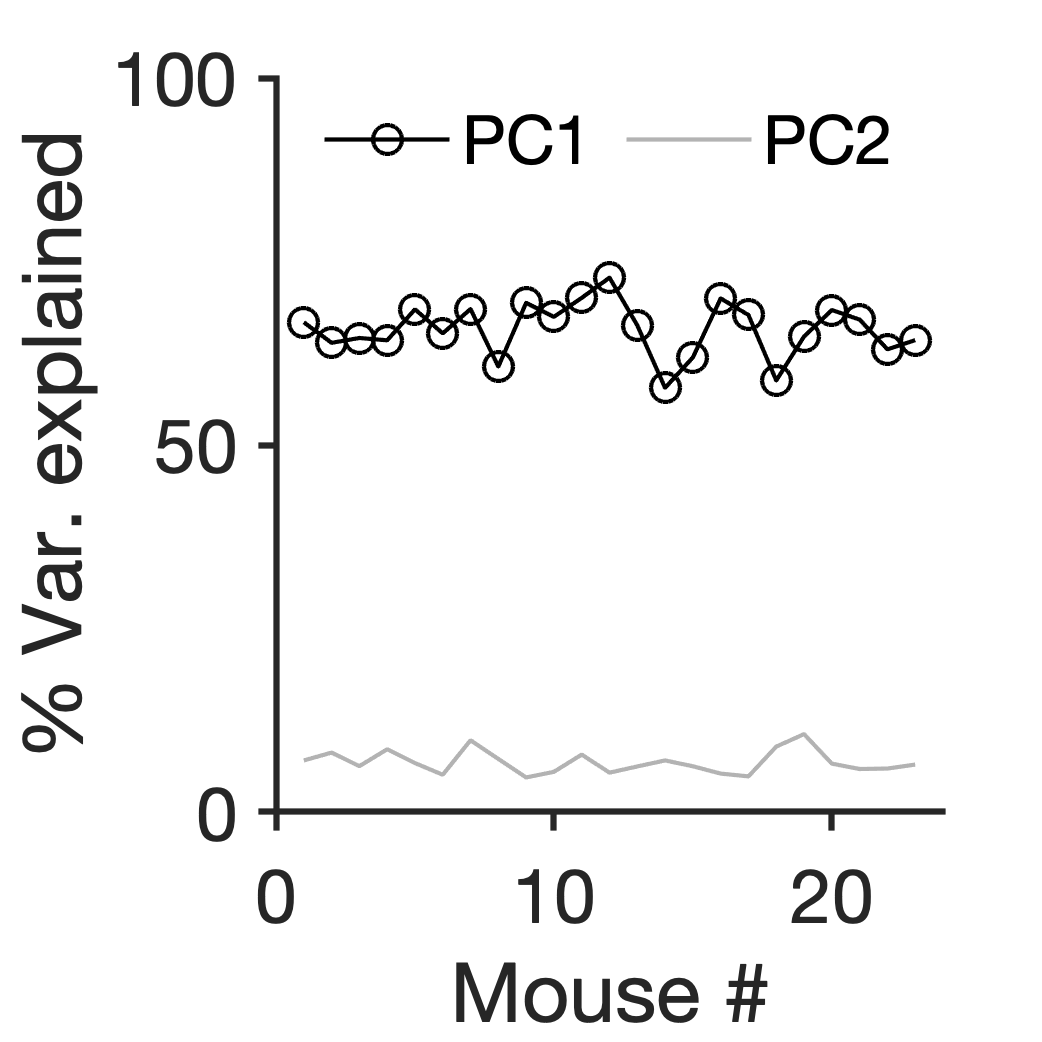

Supplement: Source code 1. [file elife-77907-code1.zip › Codes_eLife/Figure3/all/functional_connectivity/pc_space_all__13.png]

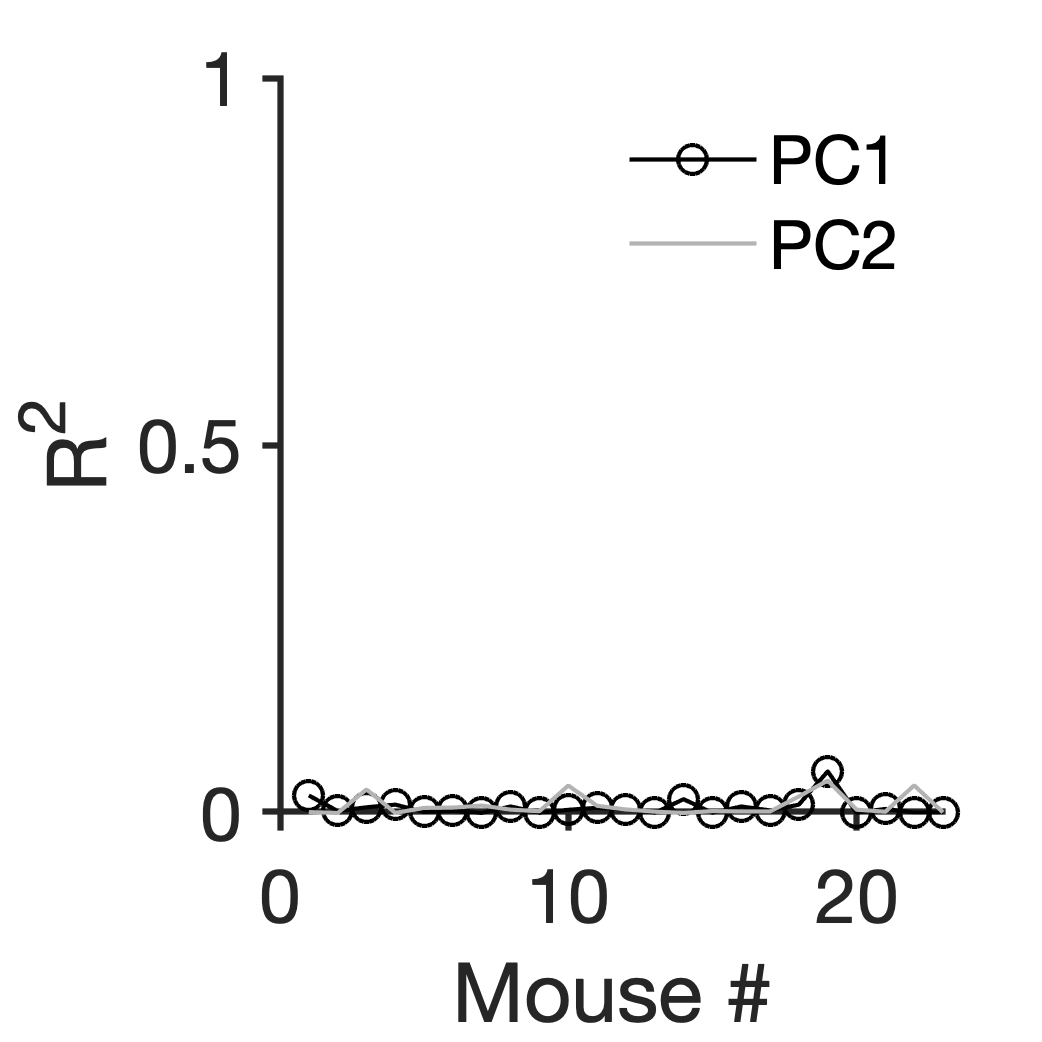

Supplement: Source code 1. [file elife-77907-code1.zip › Codes_eLife/Figure3/all/functional_connectivity/pc_space_all__12.png]

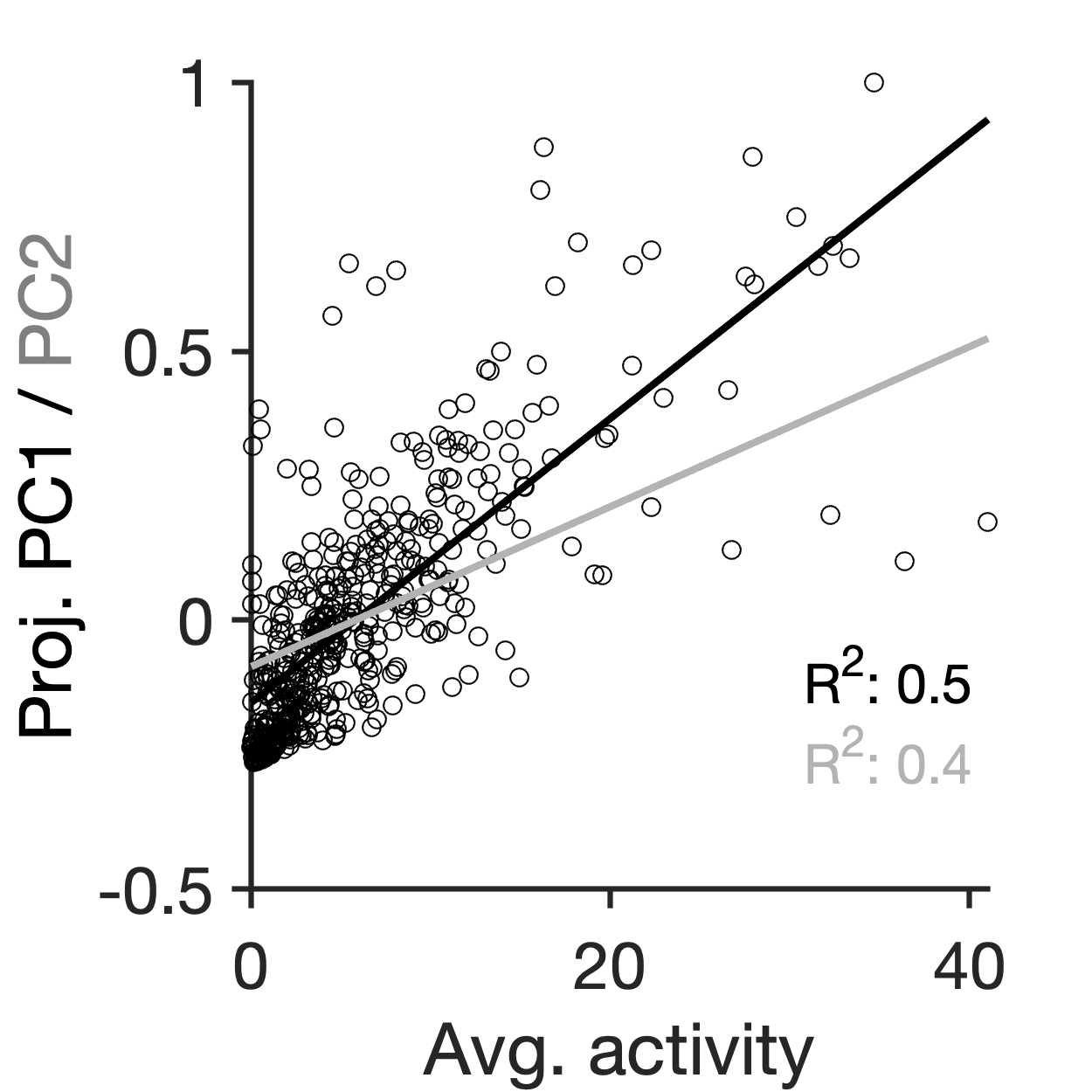

Supplement: Source code 1. [file elife-77907-code1.zip › Codes_eLife/Figure3/examples/brain_observatory_1.1/pc_space_all__23.png]

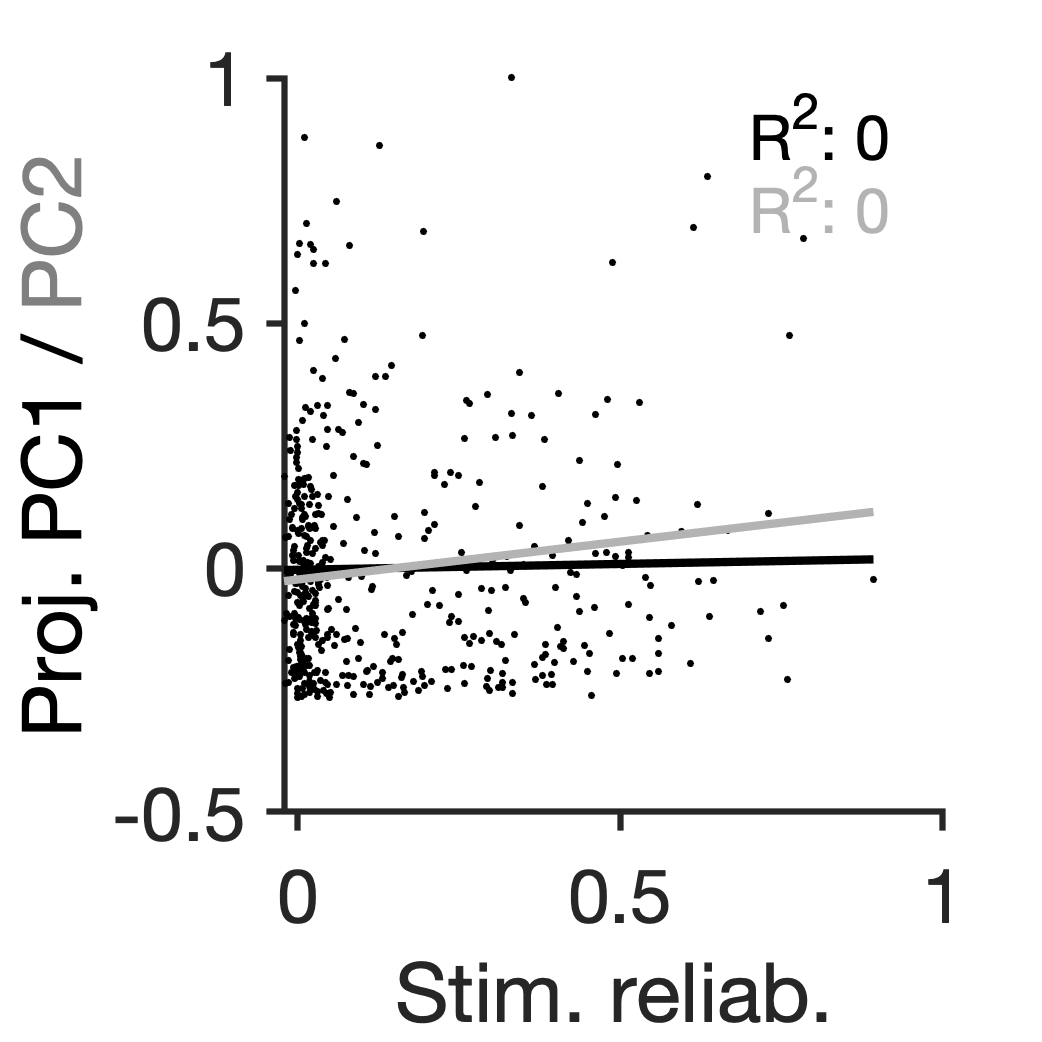

Supplement: Source code 1. [file elife-77907-code1.zip › Codes_eLife/Figure3/examples/brain_observatory_1.1/pc_space_all__22.png]

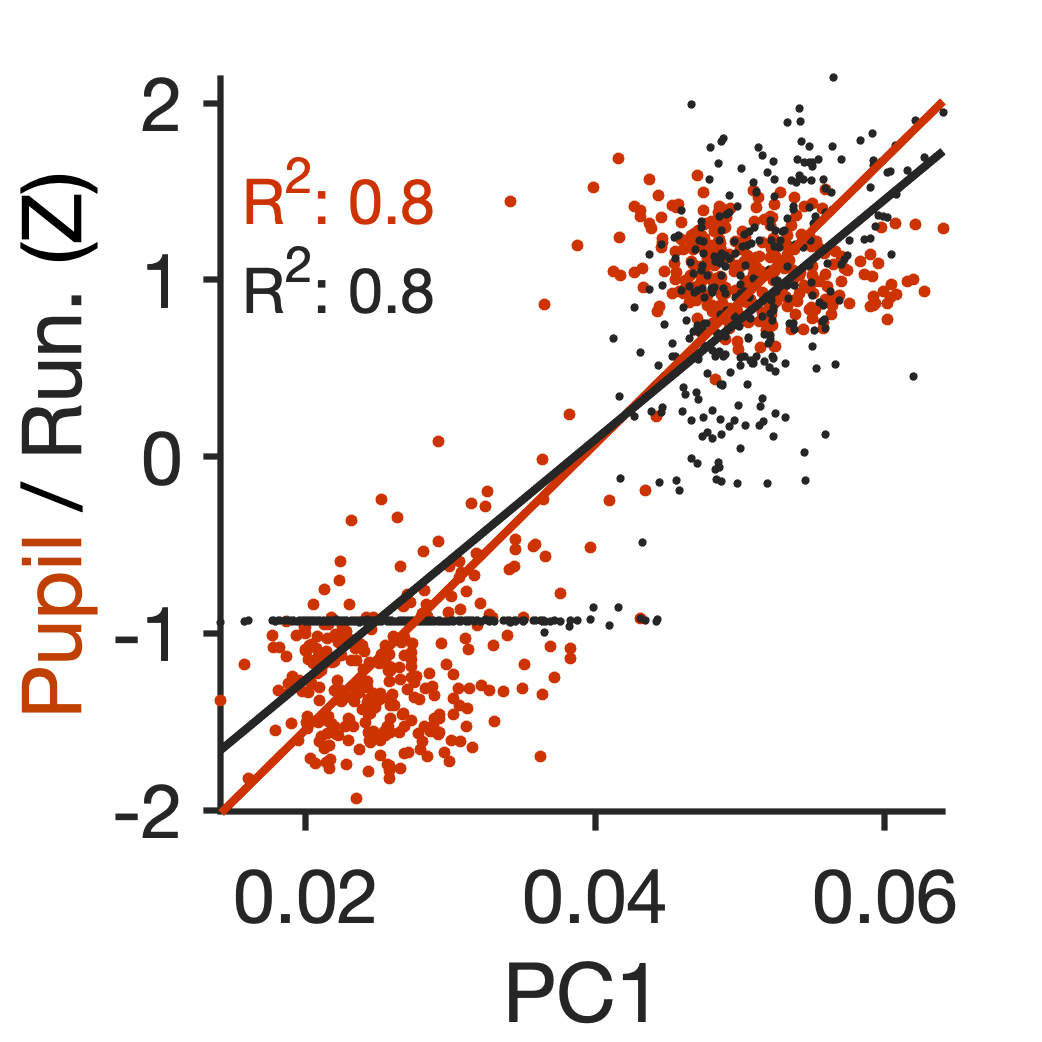

Supplement: Source code 1. [file elife-77907-code1.zip › Codes_eLife/Figure3/examples/brain_observatory_1.1/pc_space_all__21.png]

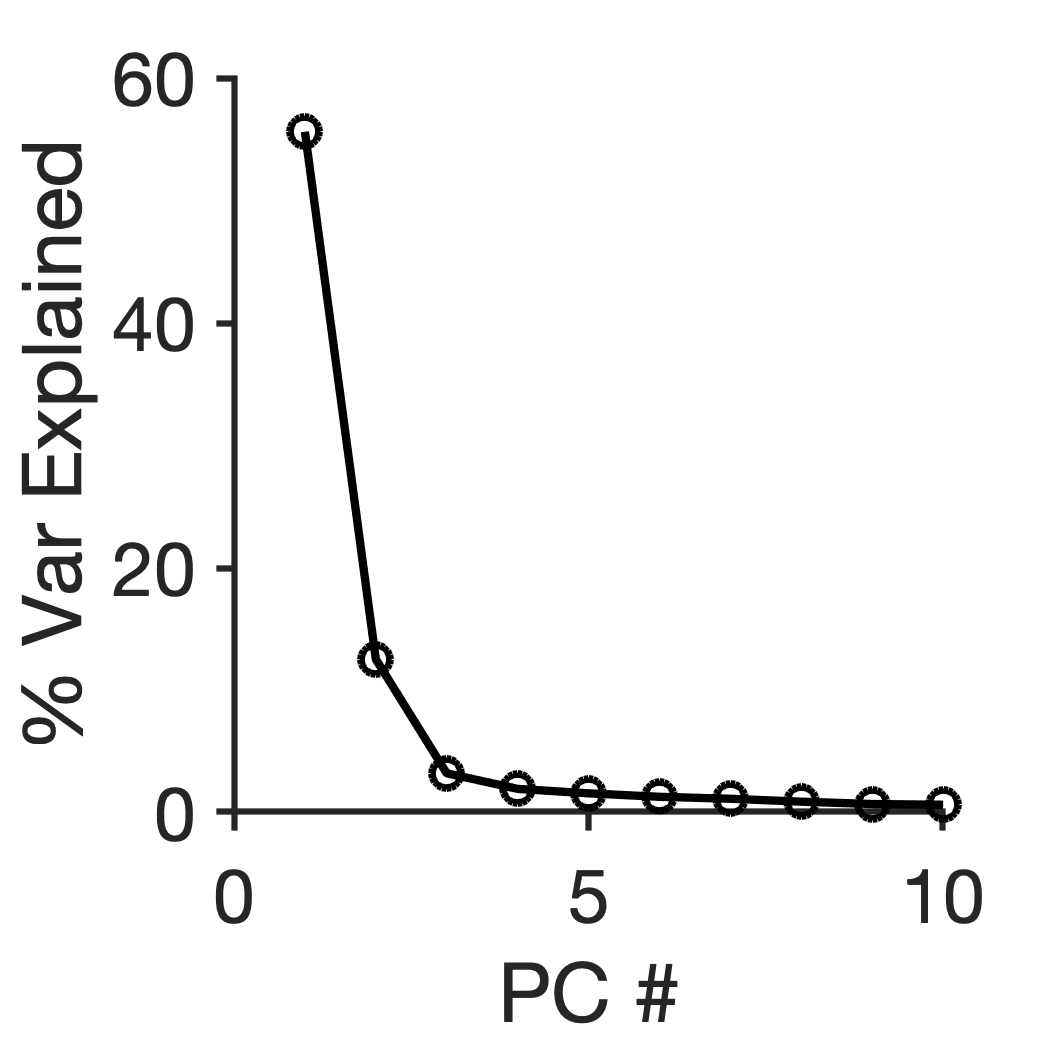

Supplement: Source code 1. [file elife-77907-code1.zip › Codes_eLife/Figure3/examples/brain_observatory_1.1/pc_space_all__11.png]

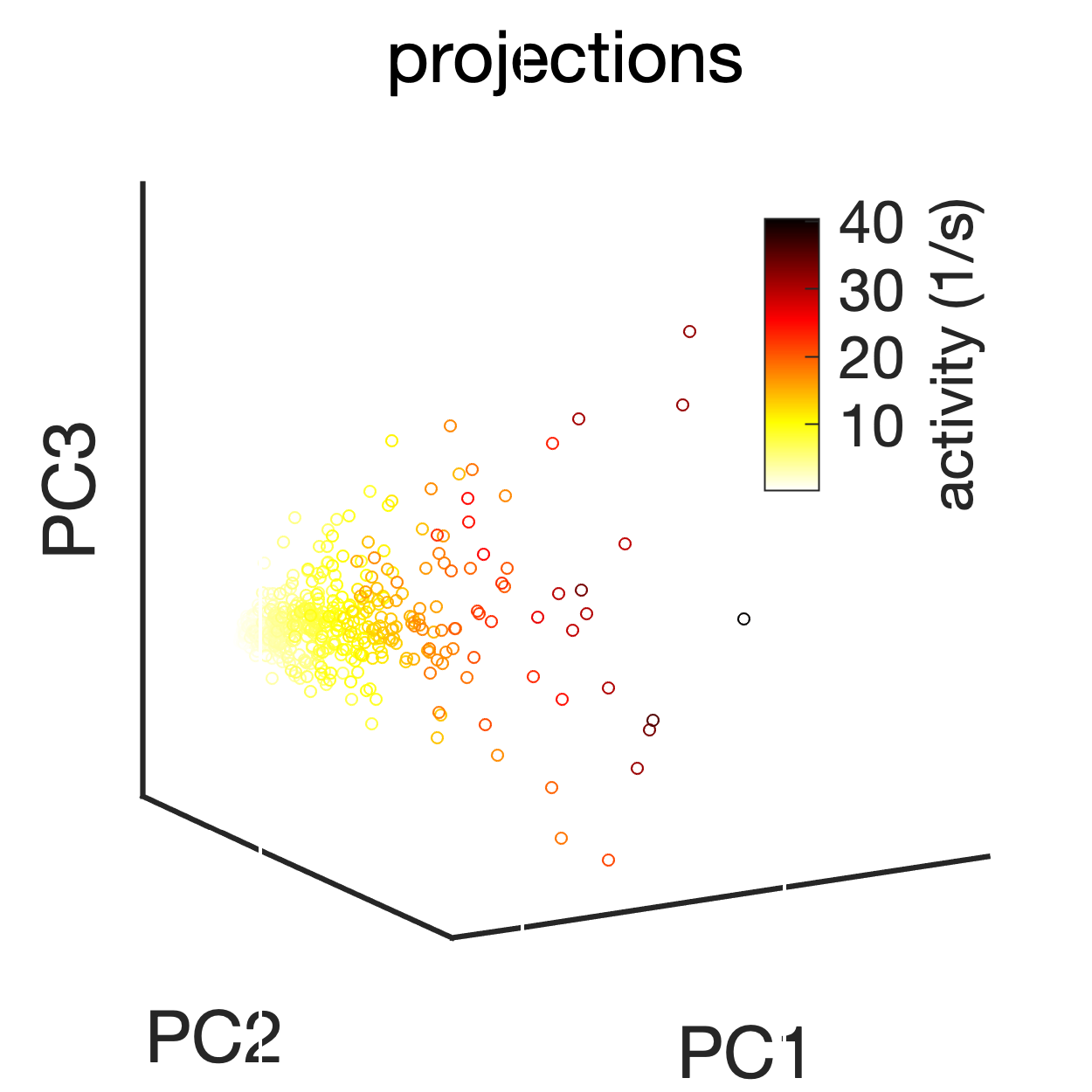

Supplement: Source code 1. [file elife-77907-code1.zip › Codes_eLife/Figure3/examples/brain_observatory_1.1/pc_space_all__13.png]

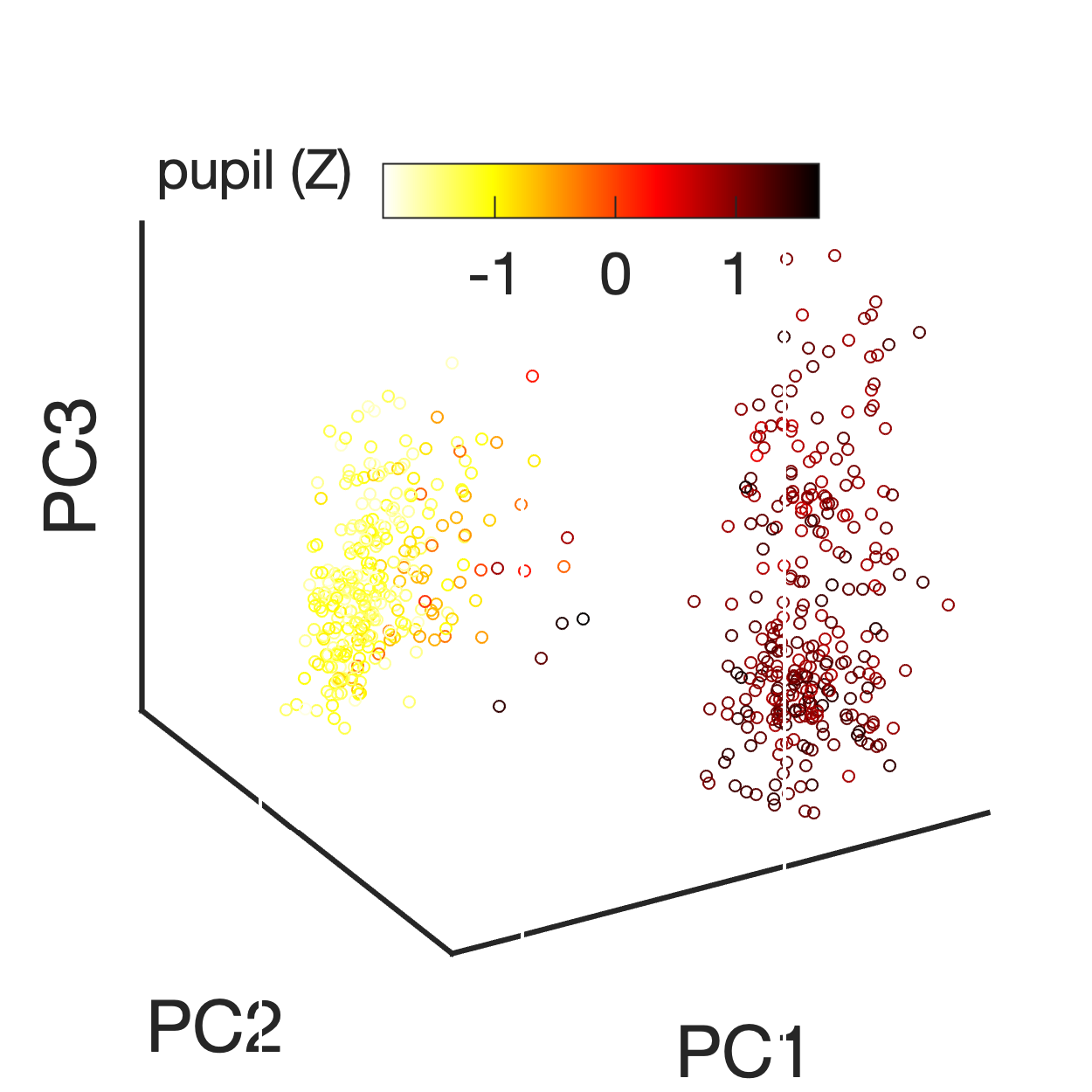

Supplement: Source code 1. [file elife-77907-code1.zip › Codes_eLife/Figure3/examples/brain_observatory_1.1/pc_space_all__12.png]

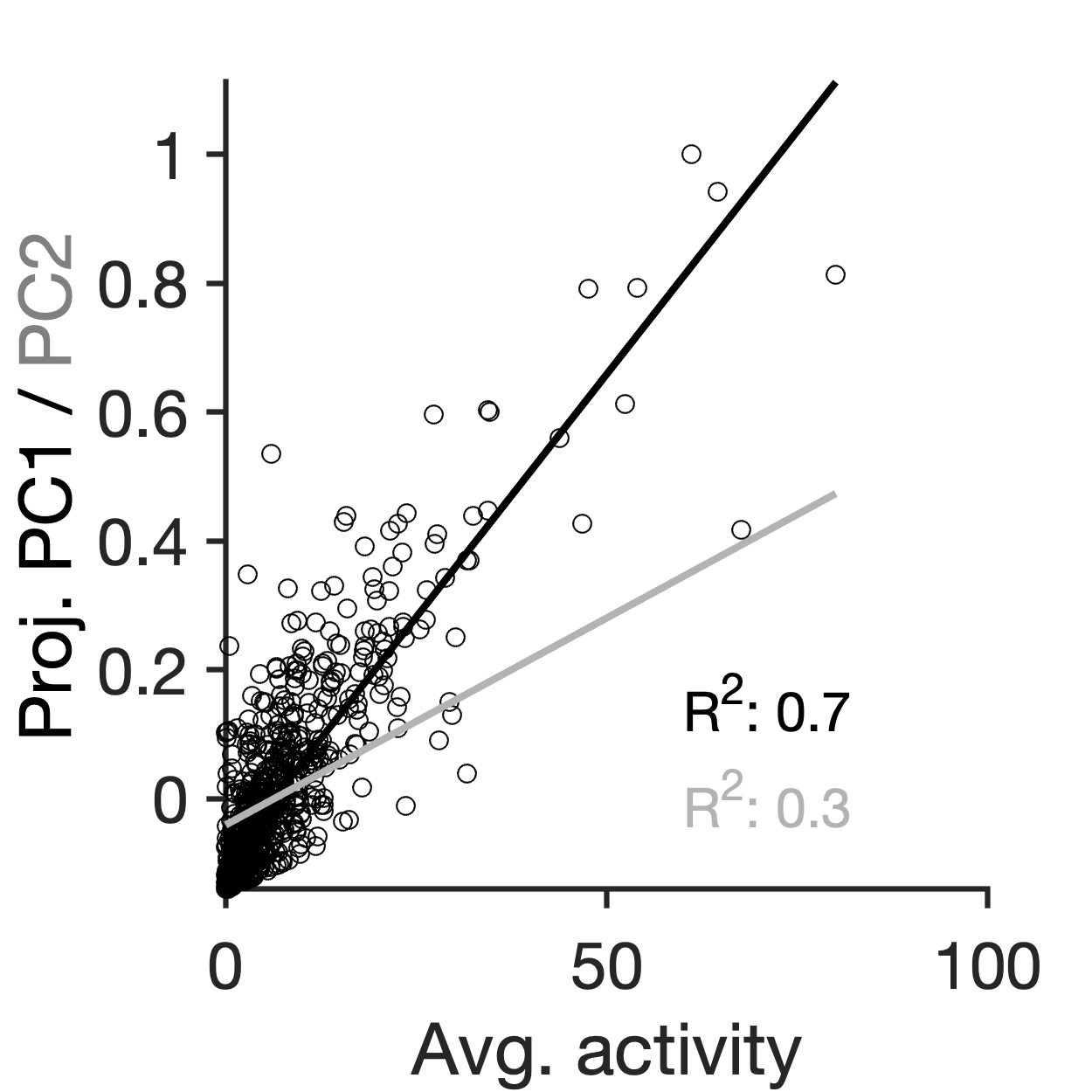

Supplement: Source code 1. [file elife-77907-code1.zip › Codes_eLife/Figure3/examples/functional_connectivity/pc_space_all__23.png]

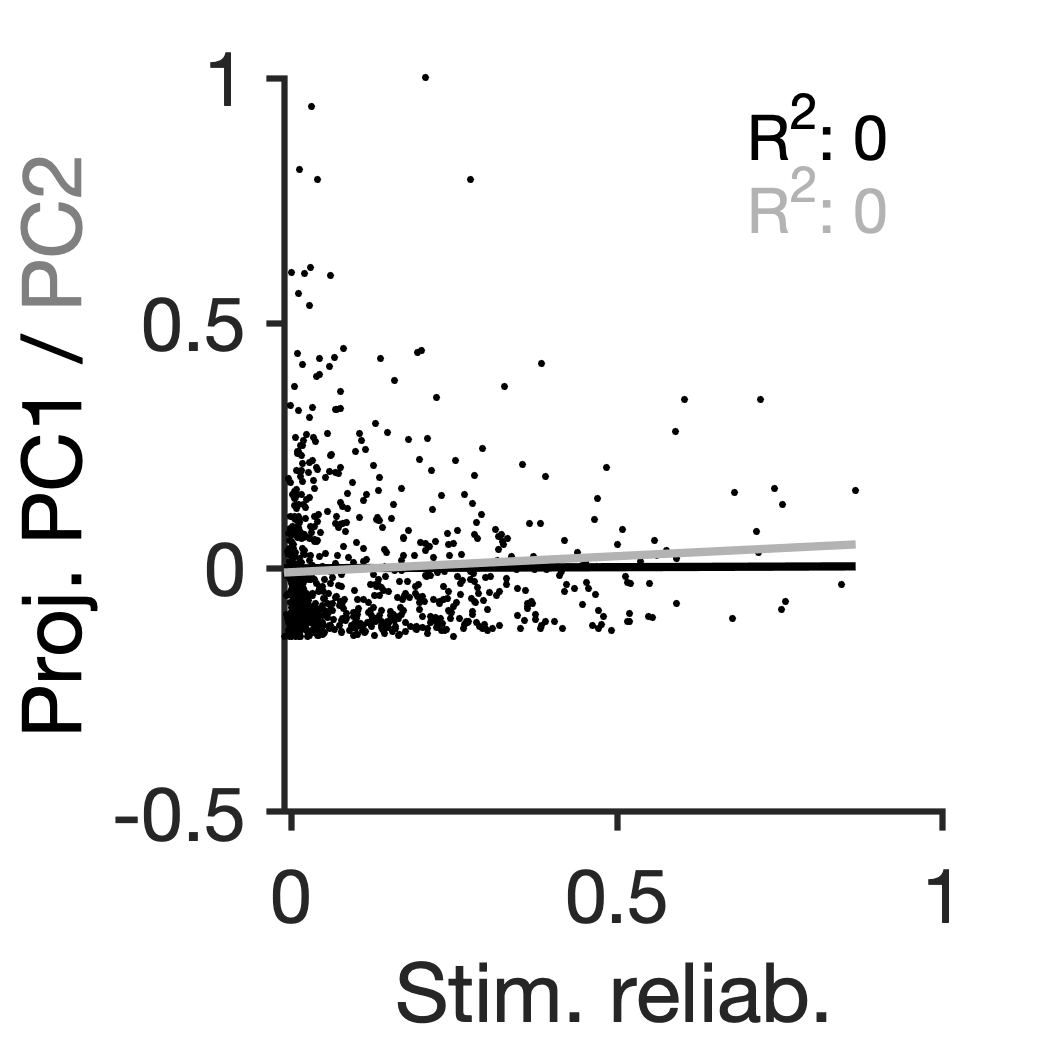

Supplement: Source code 1. [file elife-77907-code1.zip › Codes_eLife/Figure3/examples/functional_connectivity/pc_space_all__22.png]

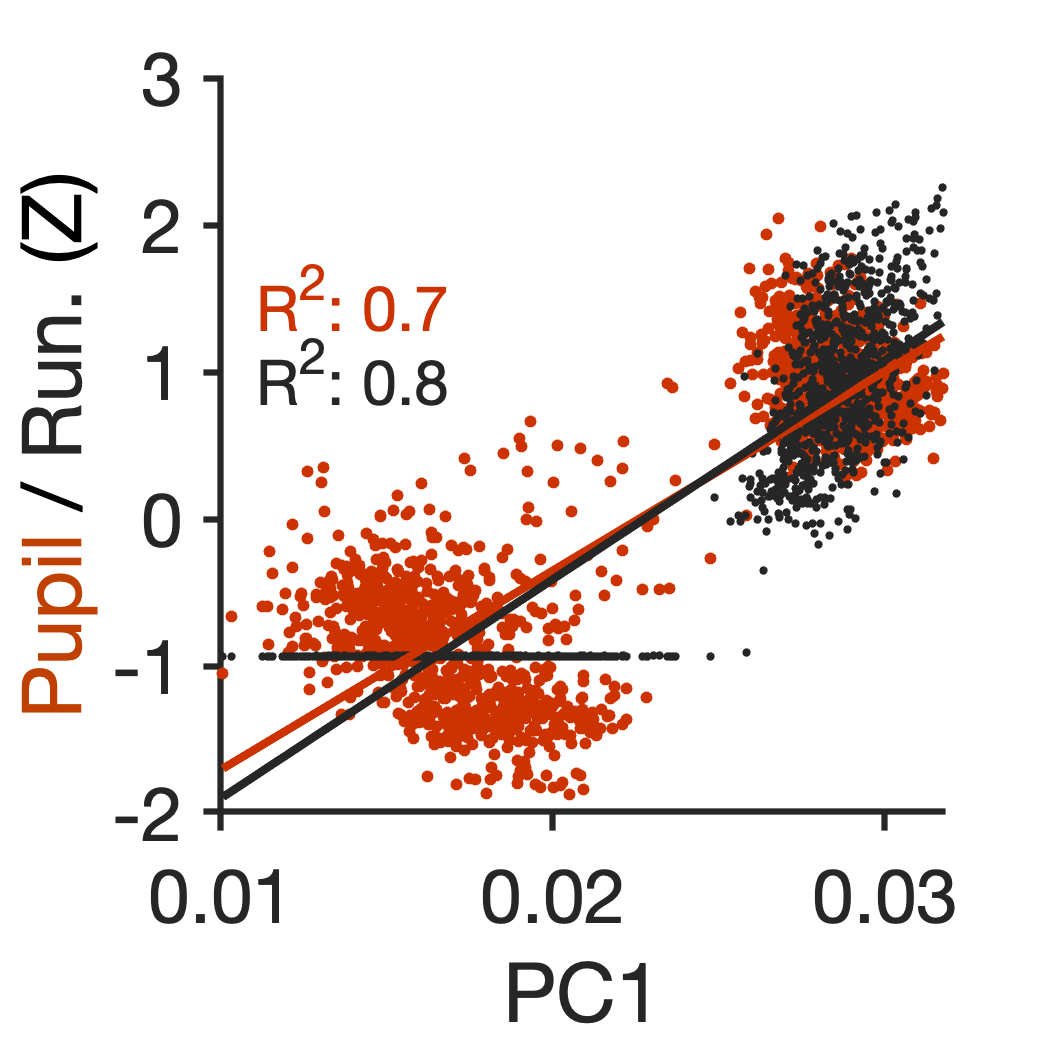

Supplement: Source code 1. [file elife-77907-code1.zip › Codes_eLife/Figure3/examples/functional_connectivity/pc_space_all__21.png]

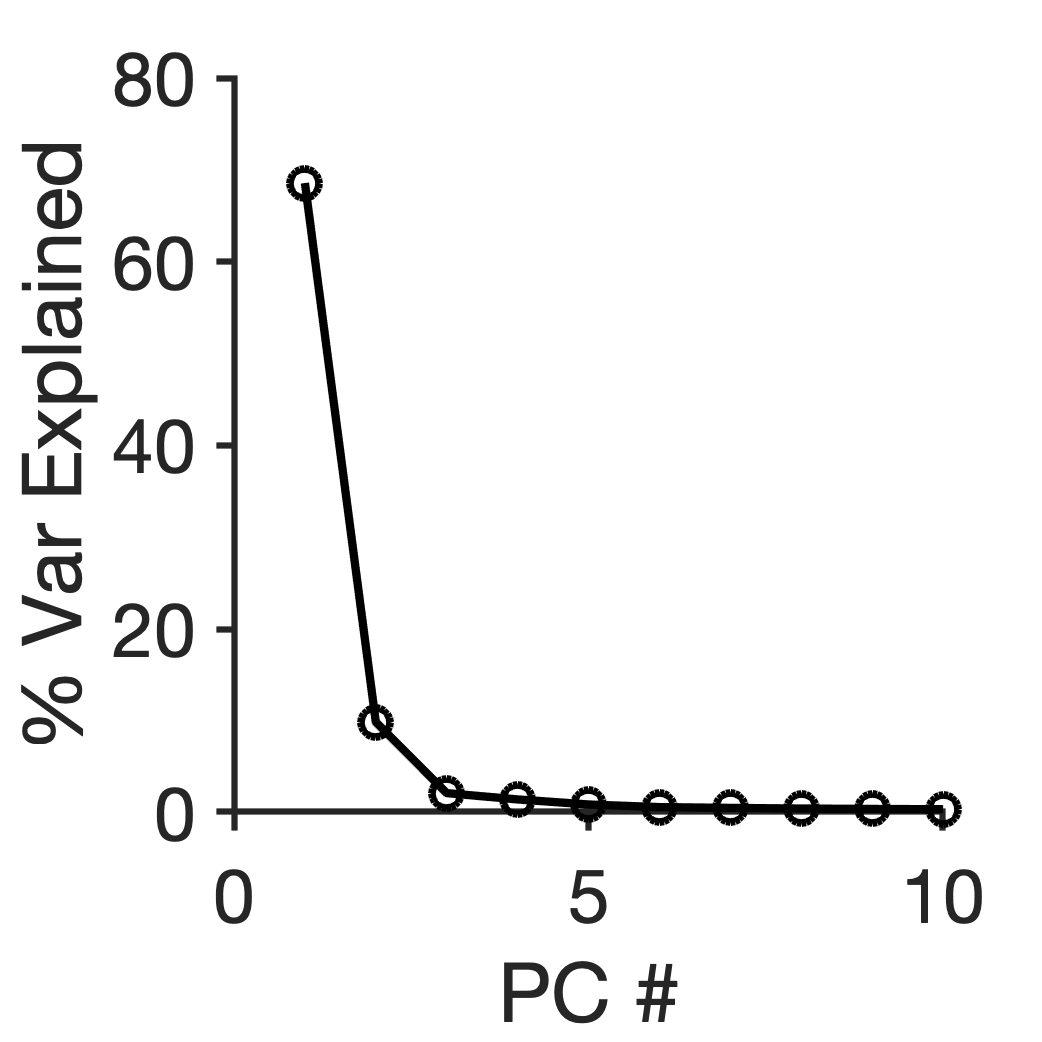

Supplement: Source code 1. [file elife-77907-code1.zip › Codes_eLife/Figure3/examples/functional_connectivity/pc_space_all__11.png]

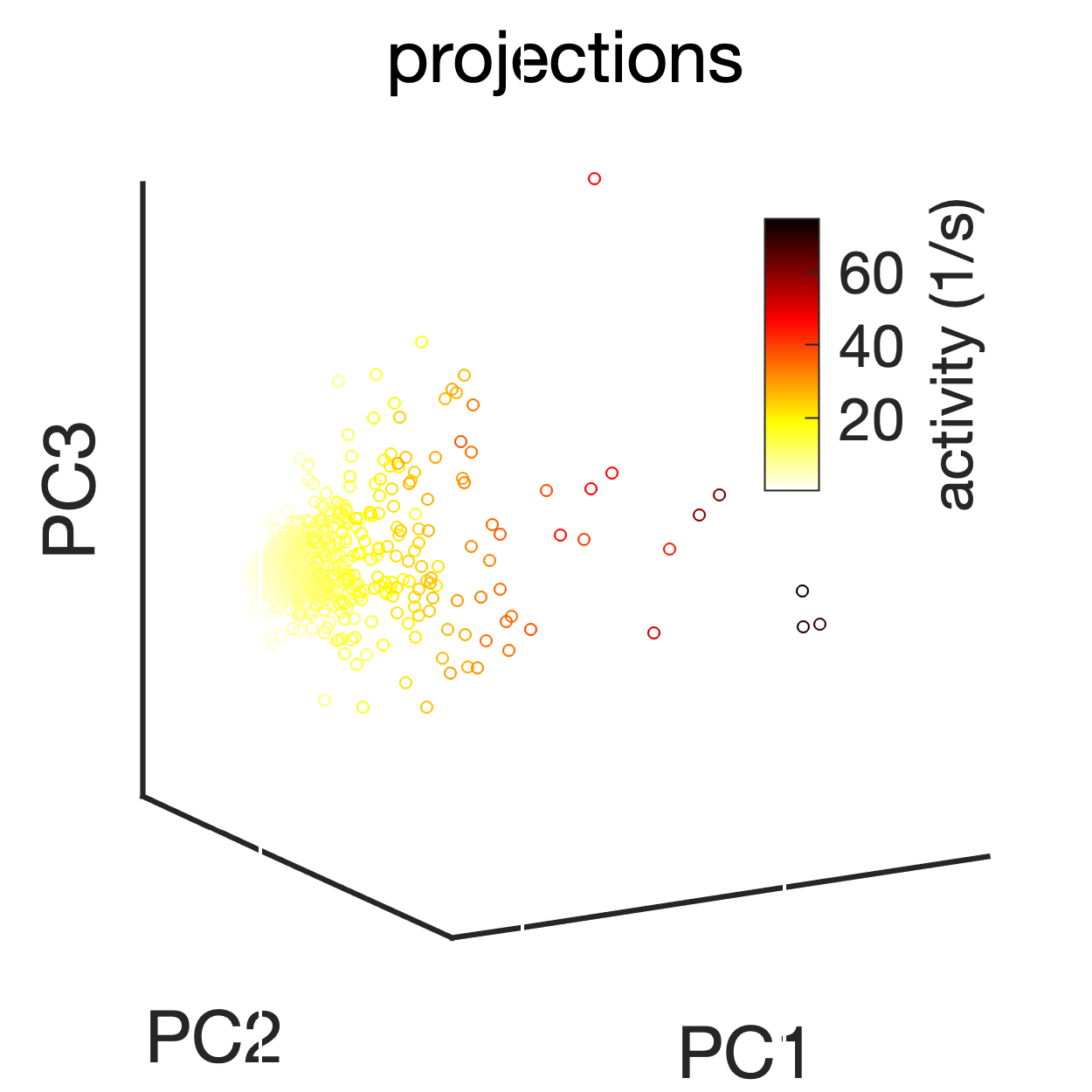

Supplement: Source code 1. [file elife-77907-code1.zip › Codes_eLife/Figure3/examples/functional_connectivity/pc_space_all__13.png]

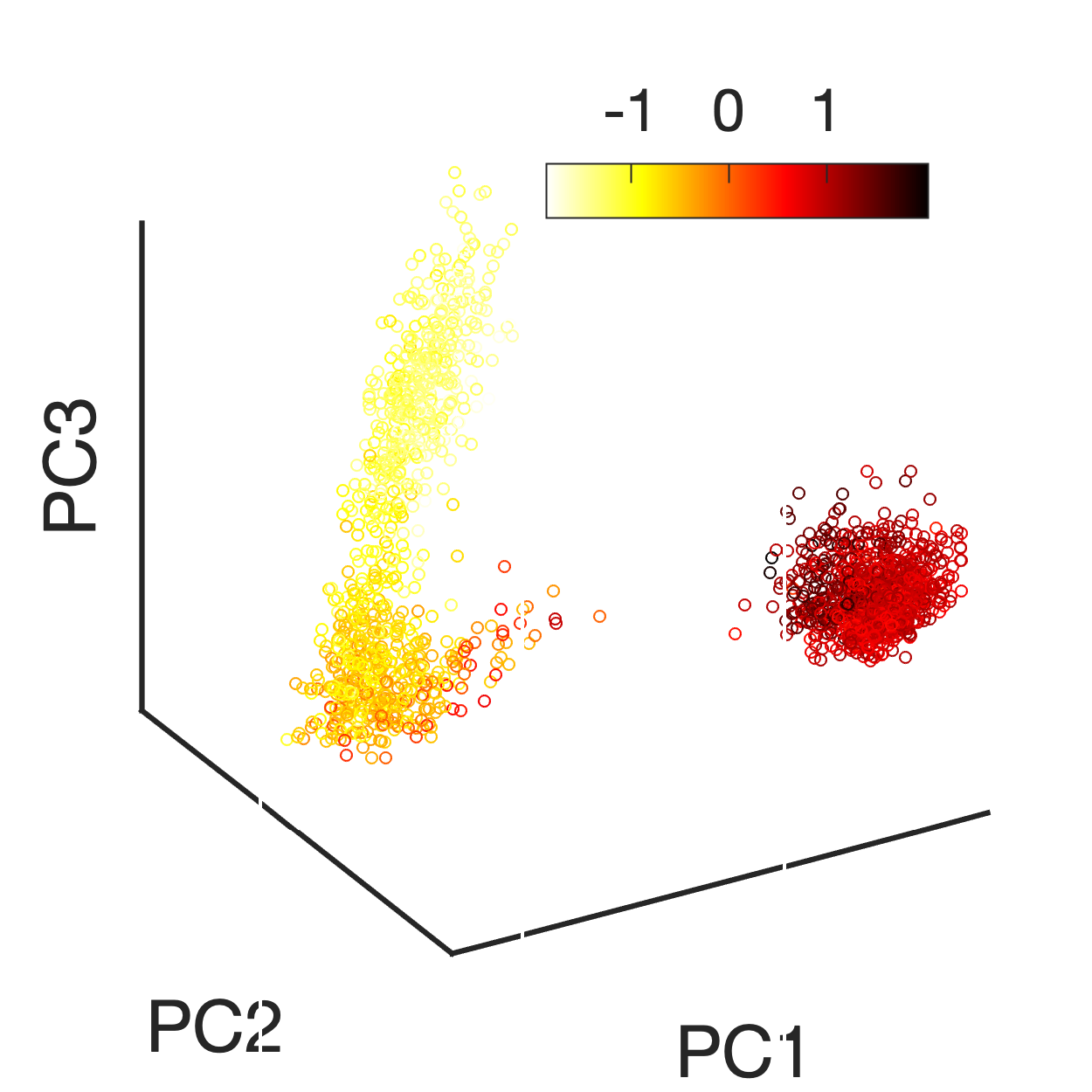

Supplement: Source code 1. [file elife-77907-code1.zip › Codes_eLife/Figure3/examples/functional_connectivity/pc_space_all__12.png]
